# RANK promotes colorectal cancer migration and invasion by activating the Ca^2+^-calcineurin/NFATC1-ACP5 axis

**DOI:** 10.1038/s41419-021-03642-7

**Published:** 2021-04-01

**Authors:** Qian Liang, Yun Wang, Yingsi Lu, Qingqing Zhu, Wenlin Xie, Nannan Tang, Lifen Huang, Tailai An, Di Zhang, Anqi Yan, Shaoyu Liu, Liping Ye, Chengming Zhu

**Affiliations:** 1 Scientific Research Center, The Seventh Affiliated Hospital, Sun Yat-sen University, Shenzhen, China; 2 Department of Pathology, The Seventh Affiliated Hospital, Sun Yat-sen University, Shenzhen, China; 3 Division of Hematology/Oncology, Department of Pediatrics, The Seventh Affiliated Hospital, Sun Yat-sen University, Shenzhen, China; 4 Center of Digestive Diseases, The Seventh Affiliated Hospital, Sun Yat-sen University, Shenzhen, China; 5 Zhongshan School of Medicine, Sun Yat-sen University, Guangzhou, China; 6 Department of Orthopaedic Surgery, The Seventh Affiliated Hospital, Sun Yat-sen University, Shenzhen, China

**Keywords:** Metastasis, Metastasis

## Abstract

The tumor necrosis factor (TNF) receptor superfamily member 11a (TNFRSF11a, also known as RANK) was demonstrated to play an important role in tumor metastasis. However, the specific function of RANK in colorectal cancer (CRC) metastasis and the underlying mechanism are unknown. In this study, we found that RANK expression was markedly upregulated in CRC tissues compared with that in matched noncancerous tissues. Increased RANK expression correlated positively with metastasis, higher TNM stage, and worse prognosis in patients with CRC. Overexpression of *RANK* promoted CRC cell metastasis in vitro and in vivo, while knockdown of *RANK* decreased cell migration and invasion. Mechanistically, *RANK* overexpression significantly upregulated the expression of tartrate-resistant acid phosphatase 5 (*TRAP/ACP5*) in CRC cells. Silencing of *ACP5* in *RANK*-overexpressing CRC cells attenuated RANK-induced migration and invasion, whereas overexpression of *ACP5* increased the migration and invasion of *RANK-*silencing cells. The ACP5 expression was transcriptionally regulated by calcineurin/nuclear factor of activated T cells c1 (NFATC1) axis. The inhibition of calcineurin/NFATC1 significantly decreased ACP5 expression, and attenuated RANK-induced cell migration and invasion. Furthermore, RANK induced phospholipase C-gamma (PLCγ)-mediated inositol-1,4,5-trisphosphate receptor (IP3R) axis and stromal interaction molecule 1 (STIM1) to evoke calcium (Ca^2+^) oscillation. The RANK-mediated intracellular Ca^2+^ mobilization stimulated calcineurin to dephosphorylate NFATC1 and induce NFATC1 nuclear translocation. Both blockage of PLCγ-IP3R axis and STIM1 rescued RANK-induced NFATC1 nuclear translocation, ACP5 expression, and cell metastasis. Our study revealed the functional expression of RANK in human CRC cells and demonstrated that RANK induced the Ca^2+^-calcineurin/NFATC1-ACP5 axis in the regulation of CRC metastasis, that might be amenable to therapeutic targeting.

## Introduction

The tumor necrosis factor (TNF) receptor superfamily member 11a (TNFRSF11a, also known as RANK) and its ligand TNF superfamily member 11 (also known as RANKL) were first identified as pivotal regulators of osteoclast development in the late 1990s (ref. ^1^). Membrane-bound or soluble RANKL from osteoblasts interacts with RANK-expressing pre-osteoclasts to induce osteoclast differentiation for bone remodeling^2^. Over the past two decades the RANKL/RANK axis has been identified as a critical signaling pathway involved in several mechanisms beyond bone homeostasis, most notably in cancer cell migration and bone development^3^. Abundant RANKL in the bone environment was identified early as a chemoattractant for bone-specific metastasis of epithelial tumors and melanoma that expressed RANK^4^. Luo et al.^5^ proposed that RANKL from tumor-infiltrating inflammatory cells activated RANK on prostate cancer and led to nuclear inhibitor of nuclear factor kappa-B (NF-κB) kinase subunit alpha (IKKα) activation to control cancer metastasis. Later their group found that tumor-infiltrating CD4^+^CD25^+^FOXP3^+^ T cells were a major source of RANKL production in breast cancer and stimulated breast cancer metastasis through RANKL–RANK signaling^6^. Furthermore, RANK is also involved in the pathogenesis of other solid tumors and nonsolid cancers^7^. Indeed, 89% of carcinomas assessed were RANK immunostaining positive and ~60% of cases exhibited >50% of RANK-positive cancer cells^8,9^. Studies have proven that increased RANK expression in tumors correlates with worse clinical parameters and progression^8^. The mechanisms by which RANK participates in the cancer development include bone environment-dependent effects and direct RANK signaling. Based on Stephen Paget’s seed and soil theory^10^, on the one hand, RANKL expressed by osteoblasts and stromal cells in bone is an important chemoattractant for tumor bone metastasis. On the other hand, tumor-associated factors secreted from cancer cells enhance bone resorption by RANKL–RANK-mediated osteoclast activation, which in turn releases tumor growth factors stored in the bone matrix to promote tumor progression^11^. This bone environment-dependent concept had been demonstrated in primary bone tumors and metastasized bone tumors^12^. In addition, many studies have indicated that direct RANK signaling has complex effects on malignancies^12^. Activation of RANK can recruit TNF receptor-associated factors to transduce canonical downstream signaling, include NF-κB, phosphatidylinositol-4,5-bisphosphate 3-kinase, mitogen-activated kinase, and C-Jun N-terminal kinase^8^. The RANK-mediated signaling network is also associated with epithelial to mesenchymal transition, stemness, and metastatic genes^8,13^.

Santini et al.^9^ found that 75% of colorectal cancer (CRC) tissues were RANK-positive when detected using immunostaining. Studies had shown that RANKL–RANK-mediated bone resorption supported CRC dissemination in bone^14^, and blockage of RANKL–RANK signaling could inhibit CRC growth and the bone resorption caused by CRC in vivo^15,16^. However, the intrinsic role of RANK expressed on CRC remains unclear. In the current study, we identified that RANK expression was upregulated in CRC tissues and correlated with worse prognosis. Functional analyses demonstrated that RANK promoted the metastases of CRC cells both in vitro and in vivo. Mechanistically, RANK overexpression in CRC not only activated the canonical key downstream of RANK signaling, but also notably induced the Ca^2+^ oscillation dependent of phospholipase C-gamma (PLCγ)-mediated inositol-1,4,5-trisphosphate (IP3) receptor (IP3R) axis and stromal interaction molecule 1 (STIM1) to activate calcineurin/nuclear factor of activated T‑cells c1 (NFATC1)-tartrate-resistant acid phosphatase 5 (TRAP/ACP5) axis to promote metastasis. Our results highlighted the intrinsic role and mechanism of RANK in CRC metastasis.

## Results

### RANK was increased in CRC and associated with worse prognosis

**Fig. 1:**
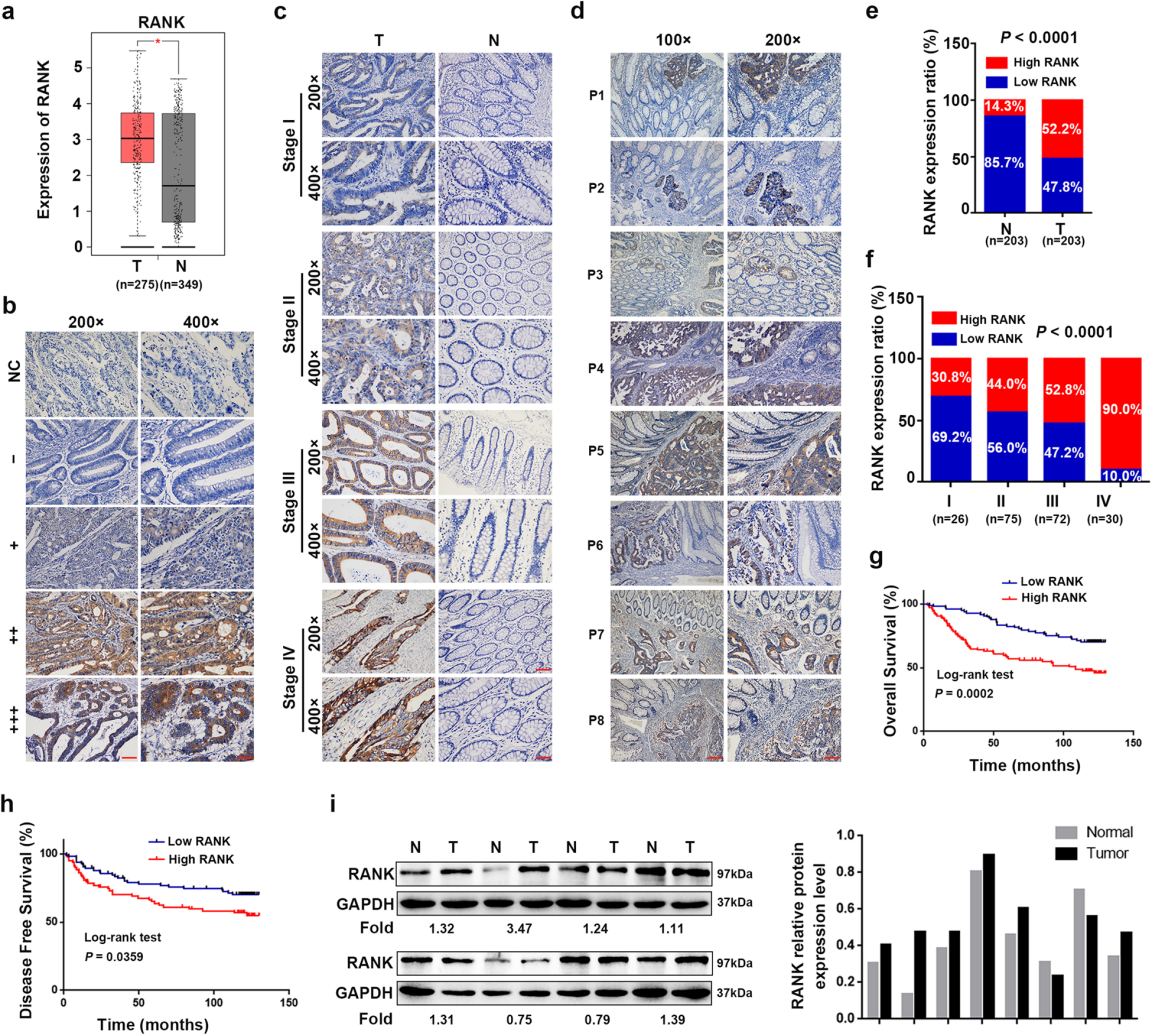
Elevated RANK expression is associated with poor survival in CRC. **a** *RANK* mRNA levels in CRC tissues and normal colorectal tissues were detected by analyzing the dataset GEPIA. *P < 0.05. **b** Representative images of different grades of RANK staining intensity were shown in CRC tissues. Staining results were classified as − (score 0), + (score 1–4), ++ (score 4–8), and +++ (score 9–12). IgG was the negative control for staining. NC negative control. **c** Representative RANK IHC staining in CRC tissues (stage I, stage II, stage III, and stage IV) and matched normal tissues far away (>10 cm) from the malignant lesion. **d** Characterization of RANK expression in CRC tissues and closely adjacent para-carcinoma tissues in eight patients (P1–P8). **e** Distribution of RANK expression in CRC tissues and matched normal tissues (*P* < 0.0001). **f** Percentage of RANK expression in CRC tumors within individual TNM stage (*P* < 0.0001). **g** Kaplan–Meier analysis of overall survival in CRC patients. **h** Kaplan–Meier analysis of disease-free survival in CRC patients. **i** Western blotting of RANK protein expressions in eight paired tumor and normal tissue samples. The tumor/normal ratios of RANK were quantified using the ImageJ software. Expression levels were normalized with GAPDH. T human CRC tissues, N paired normal colorectal tissues. Scales bars = 200 μm (100×), 100 μm (200×), and 50 μm (400×).

The GEPIA database showed that *RANK* mRNA levels were significantly increased in CRC tissues compared with those in normal colorectal tissues (Fig. 1a). Then, we used immunohistochemistry (IHC) staining to detect the expression of RANK in CRC. Representative images of different IHC staining grades of RANK are shown in Fig. 1b. RANK expression was upregulated in CRC tissues with different TNM stages compared with that in matched normal tissues distant (>10 cm) to the malignant lesion (Fig. 1c) or closely adjacent para-carcinoma tissues (Fig. 1d). As summarized in Fig. 1e, f, the proportion of high RANK expression significantly increased in CRC tissues than matched normal colonic epithelium (*P* < 0.0001) and increased gradually with the TNM stage (*P* < 0.0001). RANK expression was correlated prominently with worse clinicopathological parameters (Table 1). High RANK expression was associated markedly with decreased overall survival (OS; *P* = 0.0002) and disease-free survival (*P* = 0.0359; Fig. 1g, h). Moreover, univariate and multivariate survival analyses showed that RANK expression was an independent prognostic factor (Table 2) and recurrent factor (Table 3). Western blotting confirmed that RANK expression was upregulated in six (6/8, 75%) CRC samples compared with that in paired normal tissues (Fig. 1i). Taken together, RANK is frequently upregulated in CRC and is implicated in the pathogenesis or progression of CRC.

**Table 1:** Correlation between RANK expression and clinicopathologic features of 203 CRC patients.

Features	*N* of cases	RANK		*P* value (*χ*^2^ tests)
		Low	High	
Total				
Age (years)				
<65	126	60	66	0.952
≥65	77	37	40	
Gender				
Male	125	62	63	0.512
Female	78	35	43	
Tumor size (cm)				
<5	173	93	80	**<0.0001**
≥5	30	4	26	
CEA level (ng/ml)				
≤5	62	27	35	0.423
>5	141	70	71	
Depth of invasion				
T1	2	0	2	**0.015**
T2	32	20	12	
T3	137	68	69	
T4	32	9	23	
Lymph node metastasis				
N0	115	63	52	**0.027**
N1	62	27	35	
N2	26	7	19	
Distant metastasis				
M0	173	94	79	**<0.0001**
M1	30	3	27	
TNM stage (AJCC)				
I	26	18	8	**<0.0001**
II	75	42	33	
III	72	34	38	
IV	30	3	27	

The bold number represents the *P* values with significant differences.

**Table 2:** Univariate and multivariate analyses of prognostic parameters for overall survival in 203 CRC patients.

Prognostic parameter	Univariate analysis			Multivariate analysis		
	HR	95% CI	*P* value	HR	95% CI	*P* value
RANK expression (high vs. low)	2.323	1.480–3.647	**<0.0001**	2.144	1.357–3.387	**0.001**
Age (≥65 vs. < 65)	1.489	0.969–2.289	0.069	—	—	—
Gender (female vs. male)	1.007	0.650–1.561	0.974	—	—	—
CEA level (> 5 ng/ml vs. ≤ 5 ng/ml)	1.735	1.040–2.893	**0.035**	1.596	0.949–2.685	0.078
TNM stage (III/IV vs. I/II)	2.643	1.682–4.152	**<0.0001**	2.209	1.390–3.509	**0.001**

The bold number represents the *P* values with significant differences.

*HR* hazard ratio, *CI* confidence interval.

**Table 3:** Univariate and multivariate analyses of recurrent factors for disease-free survival in 173 CRC patients (stages I/II/III).

Prognostic parameter	Univariate analysis			Multivariate analysis		
	HR	95% CI	*P* value	HR	95% CI	*P* value
RANK expression (high vs. low)	1.718	1.036–2.848	**0.036**	1.685	1.010–2.814	**0.046**
Age (≥ 65 vs. < 65)	1.796	1.086–2.969	**0.023**	1.807	1.074–3.039	**0.026**
Gender (male vs. female)	0.943	0.564–1.576	0.822	—	—	—
CEA level (> 5 ng/ml vs. ≤ 5 ng/ml)	2.235	1.163–4.295	**0.016**	1.875	0.950–3.699	0.070
TNM stage (III vs. I/II)	1.859	1.124–3.075	**0.016**	1.764	1.040–2.994	**0.035**

The bold number represents the *P* values with significant differences.

*HR* hazard ratio, *CI* confidence interval.

### RANK promoted CRC migration and invasion in vitro and in vivo

**Fig. 2:**
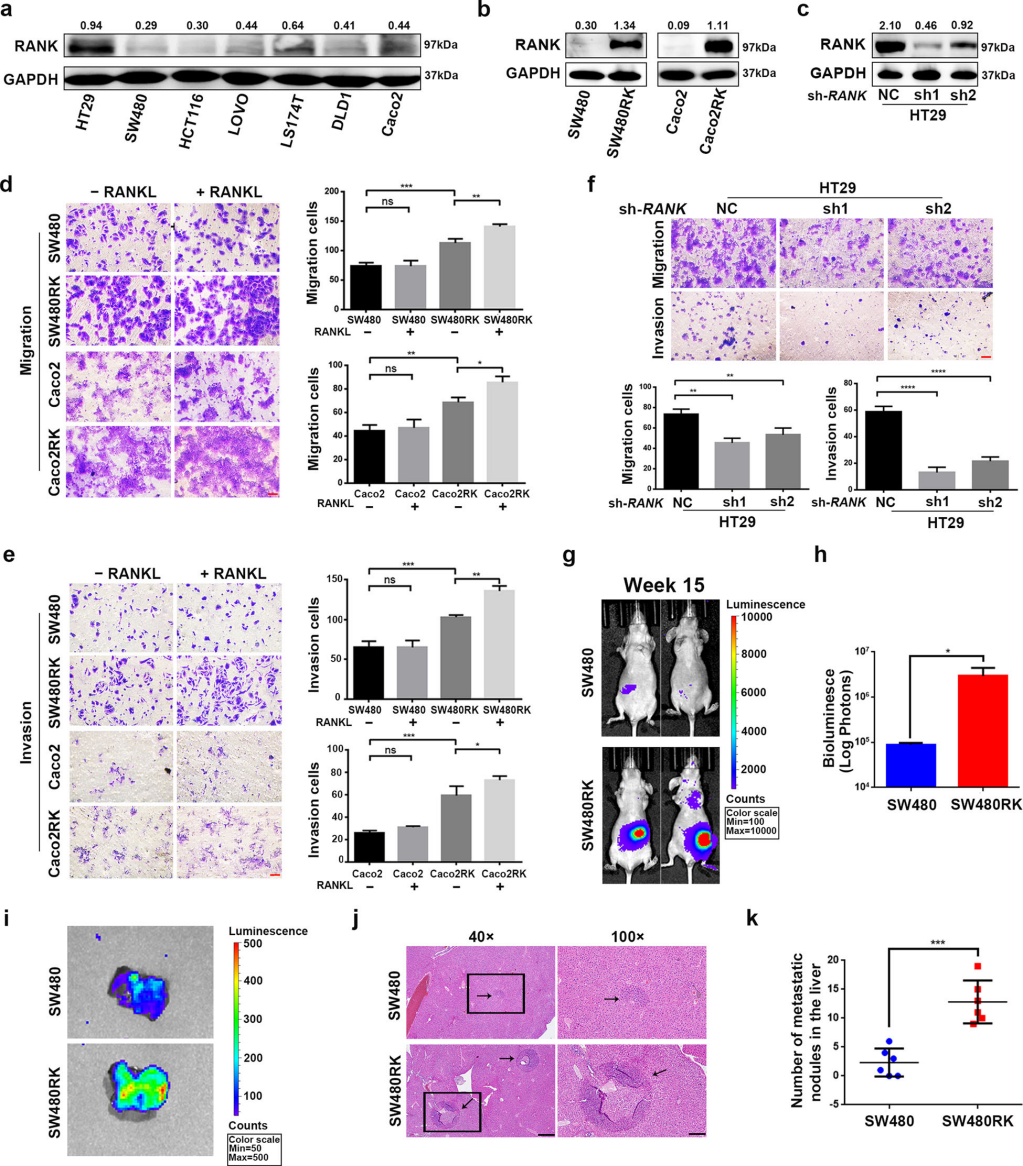
RANK promoted the metastasis of CRC cells in vitro and in vivo. **a** Protein expressions of RANK were detected by western blot analysis in seven CRC cell lines. **b**, **c** RANK stable overexpression or knockdown efficiency was confirmed by western blotting in CRC cells. **d**, **e** RANK overexpression promoted the migration and invasion of SW480 and Caco2 cells. And the addition of 100 ng/ml RANKL moderately increased the migration and invasion of *RANK*-overexpressing SW480 and Caco2 cells. Scales bars = 100 μm. **f** Knockdown of *RANK* inhibited the migration and invasion of HT29 cells. Scales bars = 100 μm. **g** Bioluminescent images of representative mice at week 15 after implantation. **h** The total tumor burden is quantified as total photons measured by bioluminescent technology. **i** Livers were excised for ex vivo bioluminescent imaging. **j** Representative results for staining of metastatic nodules (black arrows) in the livers. Scales bars = 500 μm (40×), 200 μm (100×). **k** The statistical analyses of metastatic nodules. Data are mean ± SD (*n* = 3; *n* = 6 mice/group for in vivo experiments). *P < 0.05, **P < 0.01, ***P < 0.001, ****P < 0.0001.

To investigate the role of RANK in CRC, we first screened the mRNA levels of *RANK* in CRC cell lines from the CCLE database (Fig. S1a). We then measured the RANK protein levels in seven cell lines with different mRNA levels (Fig. 2a). According to protein levels, we transfected control LV-vector and LV-RANK to construct *RANK* stably overexpressing SW480 (SW480RK) and Caco2 (Caco2RK) cells (Fig. 2b and Fig. S1b, c). Meanwhile, two independent shRNAs against *RANK* were transfected into HT29 cells to establish *RANK* stable knockdown cells (Fig. 2c and Fig. S1d). *RANK* overexpression or combined with 100 ng/ml of RANKL promoted the migration and invasion of SW480 and Caco2 cells (Fig. 2d, e), whereas knockdown of *RANK* inhibited migration and invasion of HT29 cells (Fig. 2f). However, *RANK* overexpression had no significant effect on the cell proliferation of CRC cell lines in either the absence or presence of RANKL (Fig. S1e, f). IHC staining showed that RANKL-positive cells exhibited the most similar distribution to CD3^+^CD25^+^FOXP3^+^ Treg cells in CRC tissues (Fig. S1g). The co-staining of CRC sections confirmed that RANKL and FOXP3 were colocalized (Fig. S1h) consistent with previous study^17^. We furthermore proved that RANKL was colocalized with CD4, but was not in relation to CD68^+^ macrophages, CD8^+^ killer T cells, and T helper (Th) cell subsets for analyzing their critical lineage determining transcription factors, including T-bet (Th1), GATA3 (Th2), and RORγT (Th17; Figs. S2 and S3). In addition, although the endogenous RANKL levels of CRC cells were very low (Fig. S4a), perturbation of endogenous RANKL by denosumab significantly attenuated migration and invasion of SW480RK and Caco2RK cells (Fig. S4b, c). These results indicated that baseline increase in migration and invasion was independent of exogenous RANKL, while low levels of endogenous RANKL are sufficient to elicit a metastatic phenotype in *RANK*-overexpressing CRC cells. In vivo experiments showed that mice injected with SW480RK cells developed remarkably greater local invasion and distant metastasis compared with that in the control group (Fig. 2g, h). Bioluminescent imaging and hematoxylin and eosin (HE) staining of resected livers revealed that SW40RK significantly increased liver metastatic nodules (Fig. 2i–k). These results proved that RANK functionally contributed to the migration and invasion of CRC cells in vitro and to hepatic metastasis in vivo.

### RANK regulated CRC migration and invasion by activating ACP5 expression

**Fig. 3:**
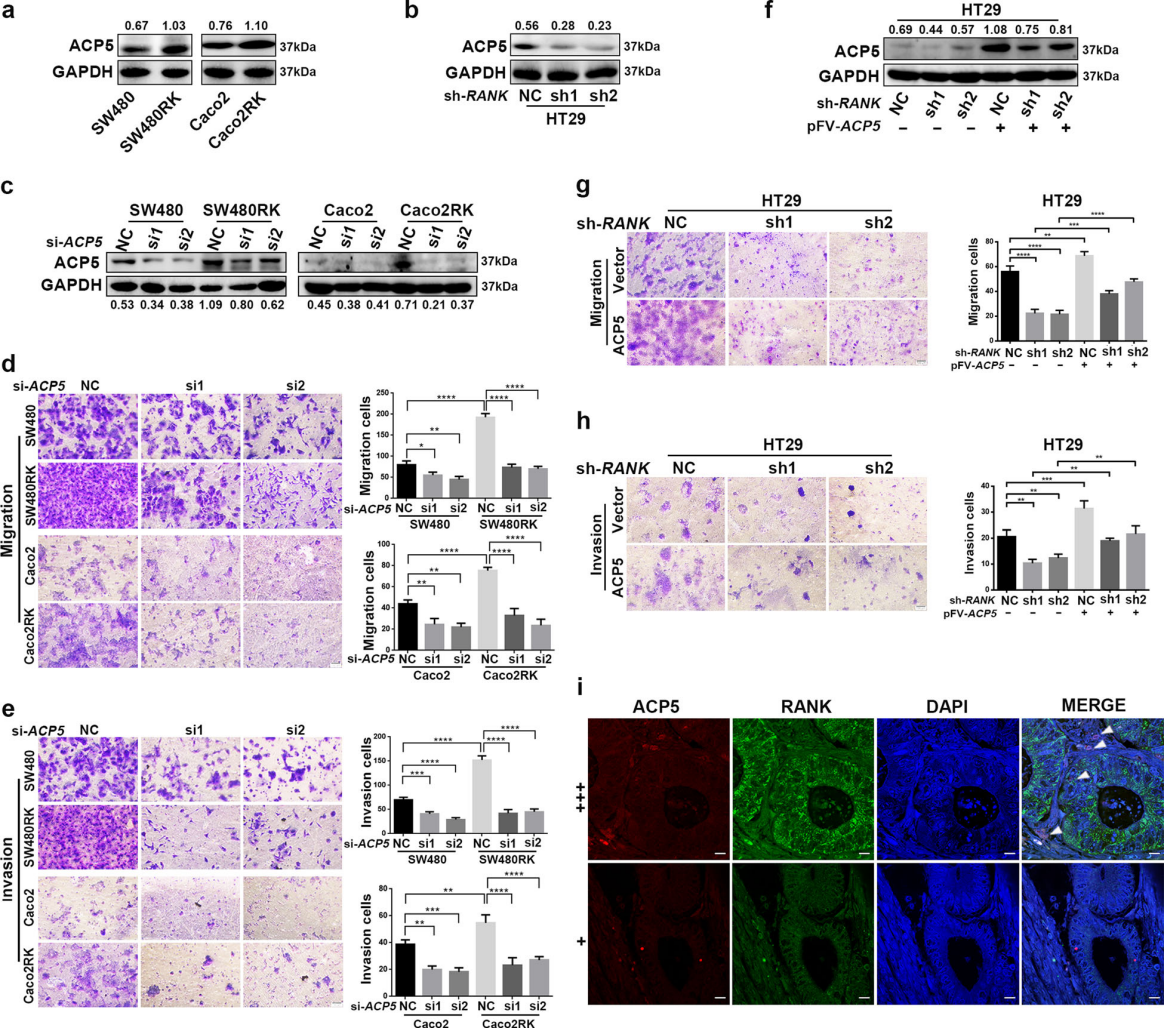
RANK regulated CRC migration and invasion by activating ACP5 expression. **a**, **b** Western blot analysis of upregulation of ACP5 protein in *RANK*-overexpressing SW480 and Caco2 cells, and downregulation of ACP5 protein in *RANK* knockdown HT29 cells. **c**, **f** Western blotting showed that successful decreased ACP5 protein in RANK overexpression CRC cells transfected with siRNA-*ACP5* and increased ACP5 protein by transfection with pFV-*ACP5* plasmid in RANK knockdown CRC cells. **d**, **e** Knockdown of *ACP5* inhibited RANK-induced migration and invasion in SW480RK and Caco2RK cells. Scales bars = 100 μm. **g**, **h** *ACP5* overexpression increased migration and invasion of RANK-silencing HT29 cells. Scales bars = 100 μm. **i** Representative immunofluorescence of ACP5 distribution in high and low RANK expression CRC tissues. Nuclei (blue) were counterstained with DAPI. The white arrowheads indicate ACP5-positive cells are distributed within the tumor and stroma with high RANK expression. + (score 1–4), +++ (score 9–12). Scales bars = 10 μm. Data are mean ± SD (*n* = 3). *P < 0.05, **P < 0.01, ***P < 0.001, ****P < 0.0001.

Increased basal levels of four canonical RANK-induced downstream pathways, phospho(p)-ERK1/2, p-P38, p-AKT, and p-NFκB-P65 with or without RANKL time-dependent stimulation were observed in SW480RK and Caco2RK cells compared with those in the parental cells (Fig. S5a, b). Furthermore, we investigated whether RANK expressed on CRC cells displayed similar downstream signaling to osteoclasts and secreted osteoclast-specific genes to degrade surrounding tissue for metastasis. Interestingly, quantitative reverse transcription polymerase chain reaction (qRT-PCR) showed that *RANK* overexpression significantly increased the mRNA levels of osteoclast marker genes, *ACP5*, *CTSK* (cathepsin K), *MMP9* (matrix metalloproteinase 9), and *VCAM1* (vascular cell adhesion molecule 1) in SW480 and Caco2 cells (Fig. S5c). Conversely, *RANK* depletion reduced the expression of these genes in HT29 cells (Fig. S5d). Moreover, *ACP5* mRNA expressions were significantly increased in CRC tissues compared with that in normal colorectal tissues (Fig. S5e), and high *ACP5* expression decreased OS from online databases (Fig. S5f). These results were confirmed by related experimental analysis of patients with CRC^18^. Thus, we were prompted to explore whether *RANK* overexpression promoted CRC migration and invasion depending on ACP5. Western blotting showed that *RANK* overexpression significantly increased ACP5 levels (Fig. 3a), whereas *RANK* depletion downregulated ACP5 levels in CRC cells (Fig. 3b). Silencing of *ACP5* in SW480RK and Caco2RK cells attenuated RANK-induced migration and invasion (Fig. 3c–e). Overexpression of *ACP5* could also rescue the reduction of HT29 cell migration and invasion induced by *RANK* silencing (Fig. 3f–h). Immunofluorescence confirmed that ACP5 distribution within the tumor and stroma was more obvious in CRC tissues with high RANK expression than in those with low RANK expression (Fig. 3i). These results indicated that ACP5 was critical for RANK-induced promotion of CRC migration and invasion.

### RANK regulated ACP5 through the calcineurin/NFATC1 axis

**Fig. 4:**
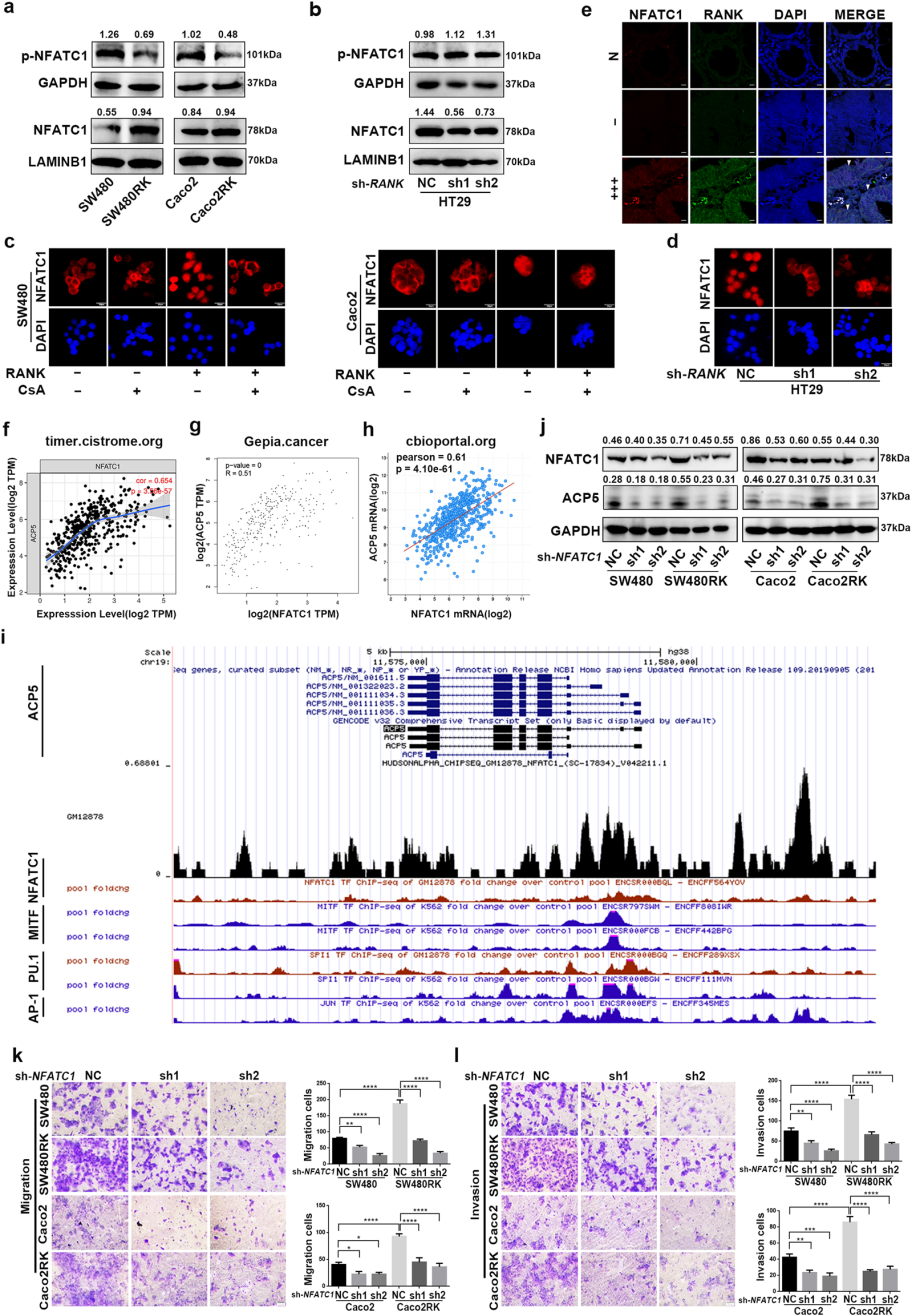
RANK upregulated ACP5 expression through driving NFATC1 nuclear translocation. **a**, **b** Western blotting analyzed the protein levels of phosphorylated NFATC1 and NFATC1 in cytoplasm, and nucleus of *RANK*-overexpressing or knockdown CRC cells. GAPDH served as the cytoplasmic control and lamin B1 as the nuclear protein loading control. **c** Immunofluorescence staining of subcellular localization of NFATC1 in RANK overexpression and control cells with or without treatment of 10 μg/ml cyclosporin A (CsA). Scales bars = 20 μm. **d** Representative immunofluorescence of subcellular localization of NFATC1 in *RANK*-depleted HT29 cells. Scales bars = 20 μm. **e** The NFATC1 subcellular localization in matched normal tissues, negative and high RANK expression of CRC tissues by immunofluorescence staining. DAPI (nuclei, blue). RANK is visualized in the membranes and in the cytoplasm surrounding the nucleus of the NFATC1 in CRC tissues (white arrowheads). − (score 0), +++ (score 9–12). N paired normal colorectal tissues. Scales bars = 10 μm. **f**–**h** Correlation analysis between mRNA levels of *NFATC1* and *ACP5* from indicated online datasets. **i** ChIP-seq data showed a UCSC Genome Browser screenshot of the *ACP5* promoter region for NFATC1 and SPI1 in GM12878 cells and MITF, SPI1, and JUN in K562 cells from ENCODE. **j** NFATC1 knockdown reversed RANK-induced ACP5 expression. **k**, **l** Silencing of *NFATC1* decreased RANK-induced migration and invasion in SW480 and Caco2 cells. Scales bars = 100 μm. Data are mean ± SD (*n* = 3). *P < 0.05, **P < 0.01, ***P < 0.001, ****P < 0.0001.

Accumulating evidence suggests that some osteoclast-specific genes, including *ACP5*, are directly regulated by NFATC1 (refs. ^19,20^). We next explored whether NFATC1 was involved in a downstream mechanism of RANK to regulate *ACP5* in CRC metastasis. High *NFATC1* expression was associated with worse OS or relapse-free survival in patients with CRC by analyzing the online databases (Fig. S6a–d). In addition, *RANK* mRNA expressions exhibited a strong positive correlation with *NFATC1* in patients with CRC in the indicated databases (Fig. S6e–g). QRT-PCR confirmed that the *NFATC1* mRNA levels were increased in SW480RK and Caco2RK cells, and decreased in *RANK* knockdown HT29 cells (Fig. S6h, i). NFATC1 undergoes efficient cytoplasmic dephosphorylation for nuclear translocation and facilitates its binding to downstream targets^21^. Western blotting demonstrated that *RANK* overexpression induced dephosphorylation of cytoplasmic NFATC1 and its nuclear translocation, while knockdown of *RANK* denoted the opposite results in CRC cells (Fig. 4a, b). Immunofluorescence also confirmed that *RANK* overexpression enriched NFATC1 levels in the nucleus, whereas *RANK* silencing had the opposite effect (Fig. 4c, d). Furthermore, increased nuclear NFATC1 levels were observed in high RANK expression CRC tissues compared with those in matched normal tissues and tumor tissues with negative RANK expression, as revealed by immunofluorescence staining (Fig. 4e). We then investigated whether RANK upregulated *ACP5* expression to promote metastasis depending on NFATC1 in CRC. The online datasets showed that *NFATC1* mRNA expressions directly correlated with *ACP5* expression in patients with CRC (Fig. 4f–h). In addition, CHIP-seq datasets from ENCODE^22^ showed that NFATC1 and other transcription factors, such as MITF, PU.1, and AP-1 were all enriched on the *ACP5* promoter (Fig. 4i). These transcription factors have been proven to interact with the *ACP5* promoter and activate its expression^19,20,23,24^. In our study, we confirmed that silencing of *NFATC1* in SW480RK or Caco2RK cells decreased *ACP5* expression significantly (Fig. 4j), and attenuated RANK-induced migration and invasion (Fig. 4k, l). These results revealed that NFATC1, activated by RANK, specifically occupied the *ACP5* promoter to regulate the migration and invasion of CRC cells.

**Fig. 5:**
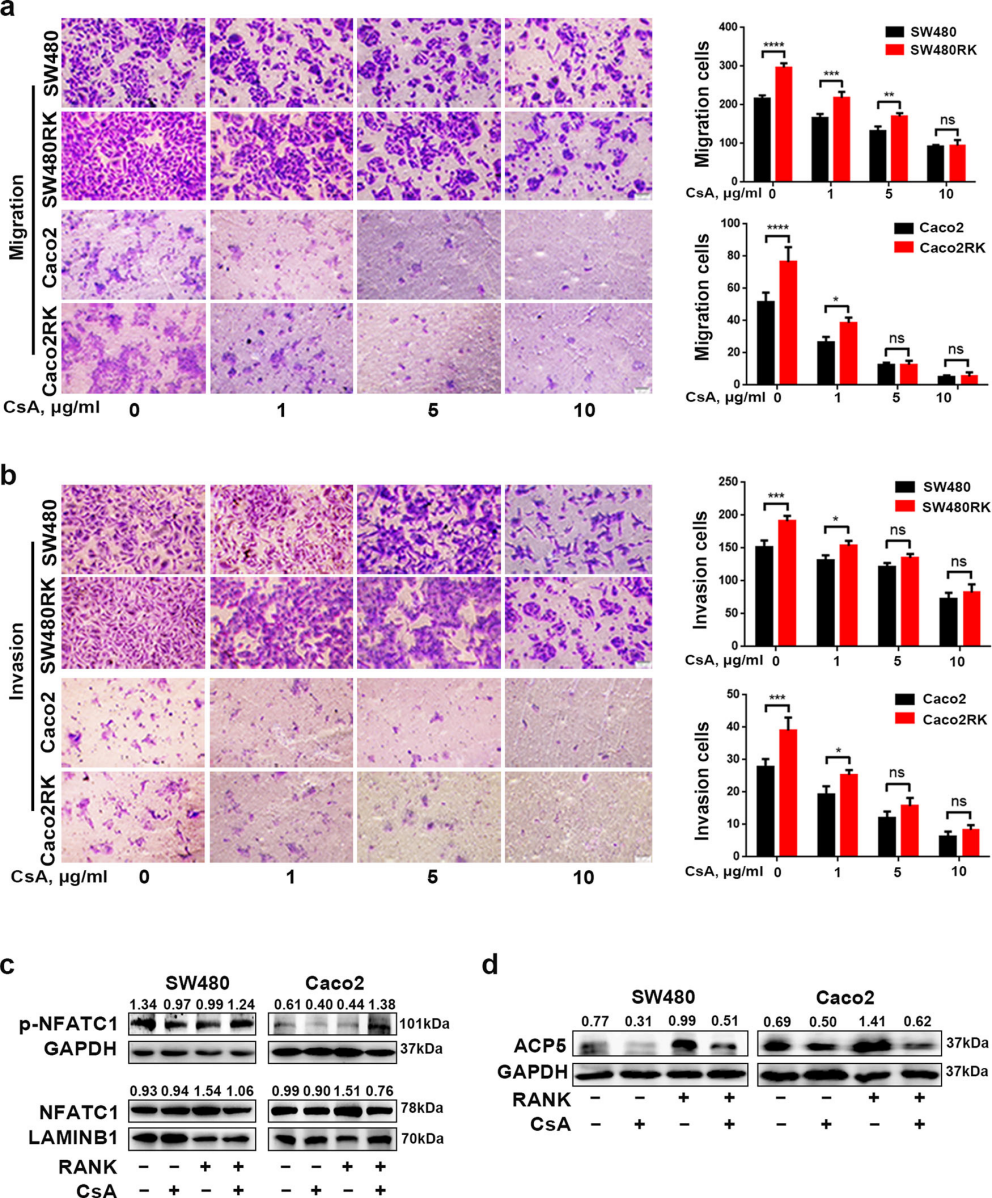
RANK-induced NFATC1-ACP5 activity was regulated by calcineurin. **a**, **b** Cyclosporin A (CsA) gradually attenuated RANK-induced migration and invasion in SW480 and Caco2 cells at 1, 5, and 10 μg/ml. Scales bars = 100 μm. **c** A total of 10 μg/ml CsA significantly decreased the nuclear translocation of NFATC1 in SW480RK and Caco2RK cells analyzed by western blotting. **d** Western blotting showed that 10 μg/ml CsA reversed the ACP5 levels in *RANK*-overexpressing CRC cells. Data are mean ± SD (*n* = 3). *P < 0.05, **P < 0.01, ***P < 0.001, ****P < 0.0001.

Notably, calcineurin, as a direct upstream regulator of NFATC1, dephosphorylates NFATC1 and initiates its nuclear translocation^21^. Thus, we further tested whether the inhibition of calcineurin had an effect on RANK-induced migration and invasion in CRC. Treatment with the calcineurin inhibitor cyclosporin A (CsA) gradually attenuated the migration and invasion of SW480RK and Caco2RK cells at 1, 5, and 10 μg/ml (Fig. 5a, b). Immunofluorescence and western blotting and showed that 10 μg/ml CsA decreased NFATC1 nuclear translocation significantly in SW480RK and Caco2RK cells (Figs. 4c and 5c). In addition, 10 μg/ml CsA reversed the upregulation of ACP5 protein levels in SW480RK and Caco2RK cells (Fig. 5d). Collectively, our results demonstrated that *RANK* overexpression activated *ACP5* expression through calcineurin/NFATC1 signaling, which eventually induced metastasis in CRC.

### RANK activated the calcineurin/NFATC1 axis via Ca^2+^ oscillation

**Fig. 6:**
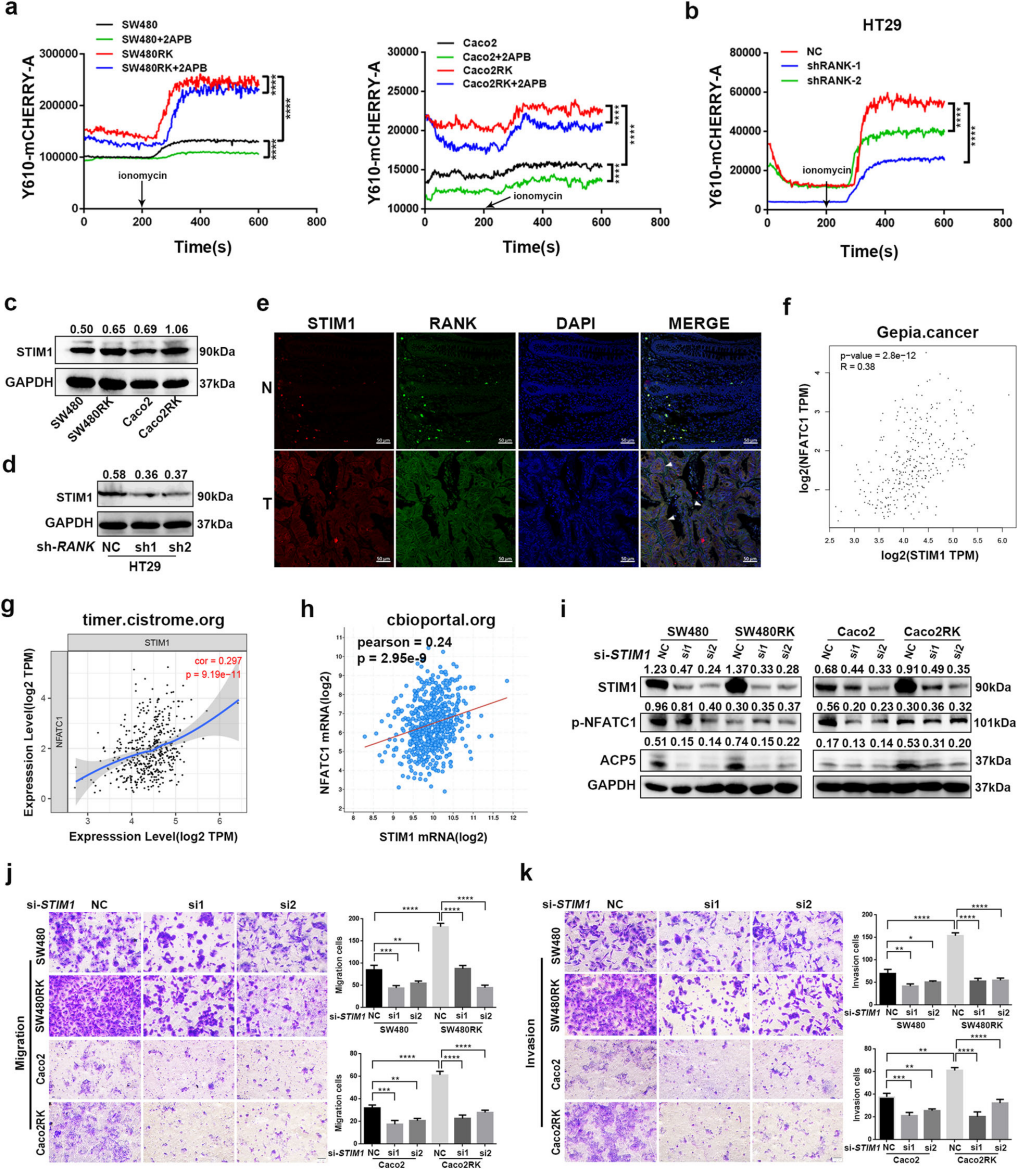
RANK activated calcineurin/NFATC1 axis by STIM1-mediated Ca^2+^ influx. **a**, **b** The calcium-flux analysis in response to ionomycin in CRC cells with *RANK* overexpression or knockdown. Besides, the effect of 100 μM 2APB on *RANK*-overexpressing SW480 and Caco2 cells were analyzed. **c**, **d** Western blotting showed that STIM1 protein levels were regulated by RANK in CRC cells. **e** STIM1 was co-stained with RANK in CRC tissue and paired normal colon tissues by immunofluorescence. The white arrowheads indicate that the overlap of RANK-positive cells and STIM1-positive CRC tissues. DAPI staining for nuclei. Scales bars = 50 μm. T human CRC tissues, N paired normal colorectal tissues. **f**–**h** The mRNA levels of *STIM1* were positively correlated with *NFATC1* in CRC assessed by online databases. **i** Silence of *STIM1* reversed RANK-induced ACP5 expression activation and increased phosphorylated NFATC1. **j**, **k** Silence of *STIM1* rescued the migration and invasion of *RANK*-overexpressing SW480 and Caco2 cells. Scales bars = 100 μm. Data are mean ± SD (*n* = 3). **P < 0.01, ***P < 0.001, ****P < 0.0001.

Ca^2+^ oscillation is essential to maintain NFATC1 in the nucleus and enables transcriptional activation of *NFATC1* (ref. ^25^). To explore the impact of Ca^2+^ signaling on RANK-induced NFATC1 activation, we first evaluated cytoplasmic Ca^2+^ oscillation in CRC cells. Calcium-flux measurement showed that overexpression of *RANK* in SW480, and Caco2 cells increased cytoplasmic Ca^2+^ levels and Ca^2+^ influx significantly (Fig. 6a). The opposite results were observed in *RANK* knockdown HT29 cells (Fig. 6b). Store-operated Ca^2+^ entry (SOCE) is a major mechanism to increase cytosolic Ca^2+^ influx and has been reported to be associated with cancer malignancy in CRC cells^26,27^. The STIM1, ORAI calcium release-activated calcium modulator 1 (ORAI1), and canonical transient receptor potential channel 1 (TRPC1) protein families are the main modulators of SOCE^27,28^. We found that the mRNA levels of *STIM1*, *ORAI1*, and *TRPC1* were upregulated significantly in SW480RK and Caco2RK cells, and downregulated in *RANK* knockdown HT29 cells (Fig. S7a, b). STIM1, as the endoplasmic reticulum (ER) Ca^2+^ sensor for SOCE, migrates from the ER to the plasma membrane to activate Ca^2+^ influx by SOCE^29,30^. STIM1 overexpression promotes CRC metastasis, and is associated with progression and poor prognosis in CRC^31,32^. Thus, we analyzed whether STIM1 was a downstream factor of RANK to regulate the calcineurin/NFATC1 axis. We found that the mRNA expressions of *RANK* correlated positively with *STIM1* in patients with CRC by analyzing database GEPIA (Fig. S7c). Western blotting confirmed that *RANK* overexpression induced STIM1 upregulation and *RANK* knockdown reduced STIM1 expression in CRC cells (Fig. 6c, d). Immunofluorescence staining further confirmed that strong cytoplasmic STIM1 staining was detected in high RANK expression CRC tissues, while weak STIM1 staining was observed in matched normal epithelial tissues with low RANK expression (Fig. 6e). Moreover, the online datasets showed that the *STIM1* mRNA levels correlated positively with *NFATC1* and *ACP5* levels in patients with CRC (Fig. 6f–h and Fig. S7d–f). Consistently, silencing of *STIM1* by small interfering RNA (siRNA) transfection inhibited the reduction of cytoplasmic phosphorylated NFATC1, and ACP5 activation induced by *RANK* overexpression (Fig. 6i). The rescue experiment revealed that silencing *STIM1* downregulated the migration and invasion of SW480RK and Caco2RK cells (Fig. 6j, k). These results indicated that the STIM1-Ca^2+^ signaling pathway is involved in the RANK-induced activation of the calcineurin/NFATC1-ACP5 axis and metastasis.

**Fig. 7:**
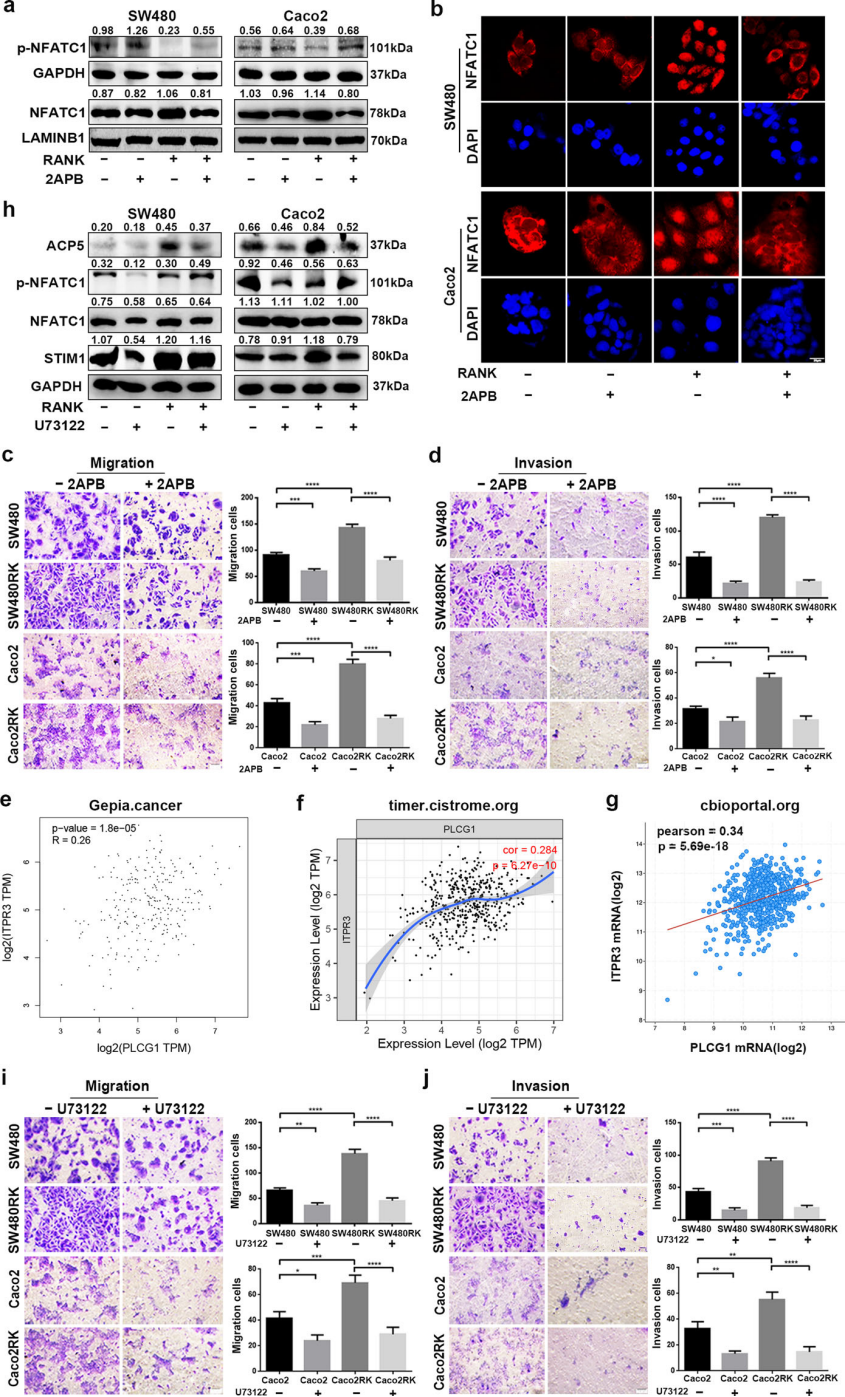
RANK induced STIM1-mediated Ca^2+^ influx and ER Ca^2+^ release by activating the PLCγ-IP3R axis. **a** Western blotting analyzed the protein levels of NFATC1 and phosphorylated NFATC1 in nuclear and cytoplasm of 100 μM 2APB-treating CRC cells. **b** Immunofluorescence staining showed nucleocytoplasmic localization of NFATC1 in CRC cells treated by 100 μM 2APB. Blue represents DAPI staining. Scales bars = 20 μm. **c**, **d** A total of 100 μM 2APB rescued RANK-mediated migration and invasion of SW480 and Caco2 cells. Scales bars = 100 μm. **e**–**g** Scatter plots showed the significant positive relationship between mRNA expressions of *IP3R* and *PLCγ* obtained by online datasets. *PLCG1*, *PLCγ*. *ITPR3*, *IP3R*. **h** A total of 10 μM U73122 rescued the protein levels of STIM1, ACP5, and the phosphorylation level of NFATC1 in SW480RK and Caco2RK cells. **i**, **j** Representative images of CRC cells treated by 10 μM U73122 subjected to the transwell migration and invasion assays. Scales bars = 100 μm. Data are mean ± SD (*n* = 3). *P < 0.05, **P < 0.01, ***P < 0.001, ****P < 0.0001.

PLCγ-mediated IP3 production and subsequent activation of ER Ca^2+^ release play a substantial role in osteoclast differentiation^33^. The indicated online datasets showed that mRNA levels of *RANK* were positively correlated with *IP3R and PLCγ* in patients with CRC (Fig. S8a–d). We supposed that RANK upregulated cytosolic Ca^2+^ concentration in CRC through ER Ca^2+^ release via PLCγ-mediated IP3 production, as well as by Ca^2+^ influx dependent on STIM1. To validate this, the effect of the IP3R antagonist, 2-aminoethoxydiphenyl-borate (2APB) on Ca^2+^ oscillation was assessed. We found that pretreatment with 100 μM 2APB could attenuate the cytosol Ca^2+^ concentration at both basal and RANK overexpression stimulatory level in CRC cells (Fig. 6a). Western blotting showed that 2APB prevented NFATC1 cytoplasmic dephosphorylation and nuclear translocation in SW480RK and Caco2RK cells (Fig. 7a). The 2APB-induced reversal of significantly nuclear translocation in *RANK*-overexpressing CRC cells was also confirmed by immunofluorescence (Fig. 7b). Furthermore, 2APB could rescue RANK-induced migration and invasion in SW480 and Caco2 cells (Fig. 7c, d). The mRNA expressions of *IP3R* were found to have positive correlation for *PLCγ* in patients with CRC by analyzing the online datasets (Fig. 7e–g). Next, we examined the effect of inhibition of PLCγ on RANK-induced activity of CRC cells. Western blotting showed that the PLCγ inhibitor U73122 reversed NFATC1 nuclear translocation, and ACP5 upregulation in SW480RK and Caco2RK cells (Fig. 7h). Notably, U73122 decreased the protein levels of STIM1 significantly in *RANK*-overexpressing CRC cells, indicating that PLCγ-mediated IP3 also regulates the activity of SOCE (Fig. 7h). The online databases also confirmed that mRNA expressions of *IP3R* correlated positively with *STIM1* in patients with CRC (Fig. S8e–g). Furthermore, U73122 significantly attenuated the migration and invasion of SW480RK and Caco2RK cells (Fig. 7i, j). The above results revealed that RANK induced the calcineurin/NFATC1 axis by activation of PLCγ-IP3-STIM1-mediated ER Ca^2+^ release and Ca^2+^ influx.

## Discussion

**Fig. 8:**
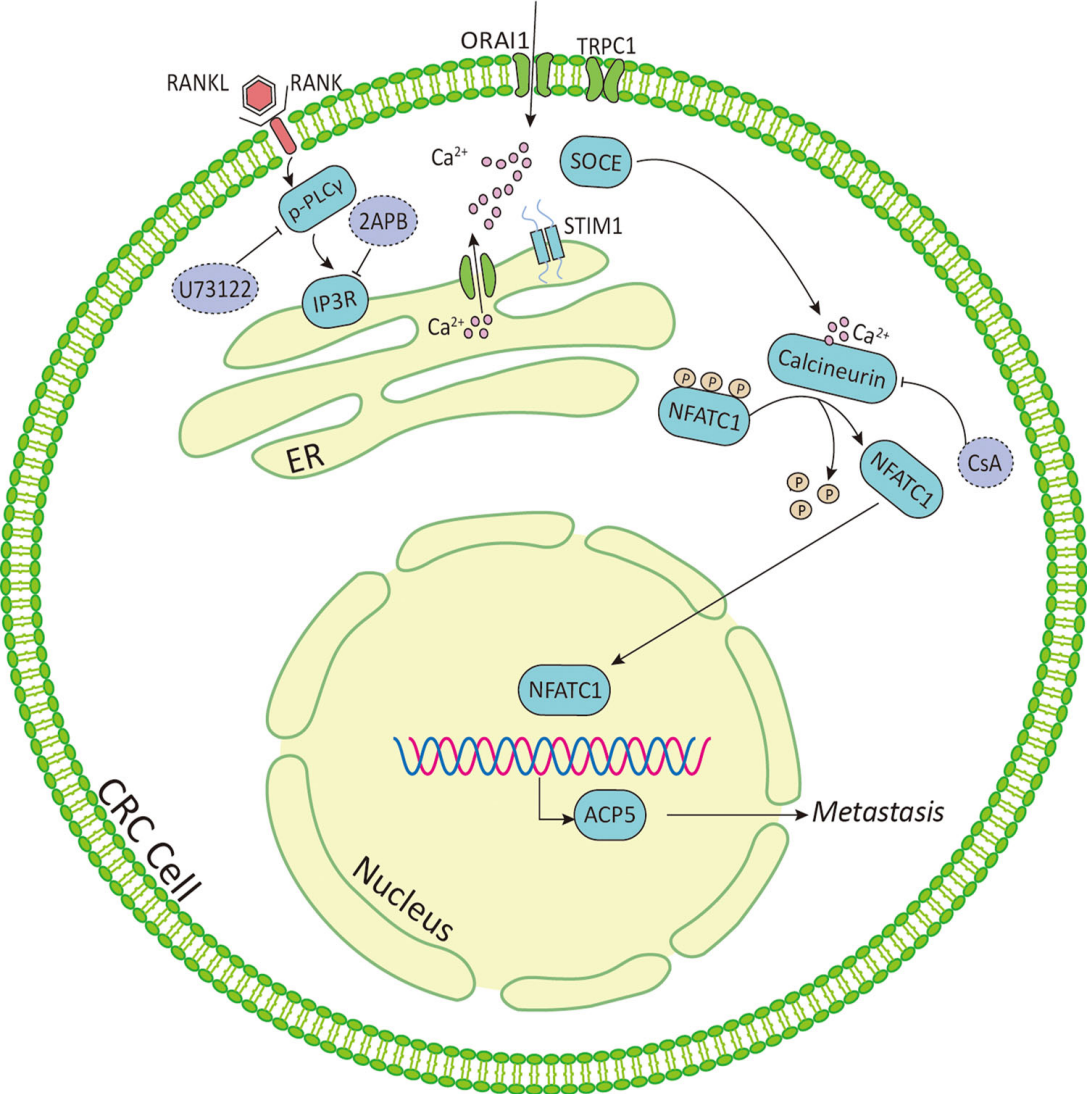
Schematic summary. Active RANK expression allows for Ca^2+^ release from ER dependent on PLCγ-mediated IP3R and Ca^2+^ influx from the extracellular milieu through STIM1-regulated SOCE. Besides, PLCγ-mediated IP3R axis act upstream of STIM1 to coactivate Ca^2+^ oscillation. The Ca^2+^ oscillation in the cytoplasm leads to calcium-dependent calcineurin activation and NFATC1 transcription of osteoclast-specific gene, *ACP5*, which promotes CRC metastasis. PLCγ phospholipase C γ, IP3R inositol-1,4,5-trisphosphate receptor, SOCE store-operated calcium entry, STIM stromal interaction molecule, TRPC transient receptor potential channel, NFATC1 nuclear factor of activated T cells c1, CsA cyclosporine A, 2APB 2-aminoethoxydiphenyl-borate, ACP5 tartrate-resistant acid phosphatase 5, ER endoplasmic reticulum.

Approximately 25% of patients with CRC present with metastases at initial diagnosis and almost 50% ultimately develop metastases, leading to the high mortality rates observed in CRC^34^. Despite improvements in therapeutic early-stage CRC over past decades, no effective therapy is currently available for advanced or metastatic CRC for poor understanding of the mechanisms underlying CRC metastasis^35^. In the present study, we found higher RANK expression in CRC tissues compared with that in matched normal tissues, which correlated with worse prognosis. Furthermore, RANK expressed in CRC-activated PLCγ-mediated IP3 signaling to evoke Ca^2+^ release from the ER, and upregulated STIM1 to promote Ca^2+^ influx through SOCE, which cooperate to generate Ca^2+^ oscillation. In addition, STIM1 was also regulated by PLCγ induction. RANK-mediated intracellular Ca^2+^ mobilization subsequently stimulated calcineurin to dephosphorylate NFATC1 and induce NFATC1 nuclear translocation. In the nucleus, NFATC1 transcriptionally upregulates osteoclast-specific gene *ACP5* to promote the migration and invasion of CRC (Fig. 8), indicating that RANK might serve as a potential therapeutic target.

To the best of our knowledge, this study is the first to report functional RANK expression in CRC. Jones et al.^4^ first mentioned the relationship between RANK and metastasis in CRC cells. Their study showed that the human CRC cell lines SW480 and Colo205 had no detectable levels of RANK. These CRC cells failed to migrate when induced by RANKL and metastasized into the bones after intracardiac injection. Thus, for a long time afterward, research associated with RANK in cancer mainly focused on breast and prostate cancer, which frequently develop bone metastasis. RANK also has a role in other cancers, such as osteosarcoma^36^ and melanoma^37^; however, there have been few studies in CRC. This might be explained by the fact that RANK is related to the bone environment, whereas CRC mainly develops liver metastasis, not bone metastasis, like breast and prostate cancer. Although one study mentioned that 75% of CRC tissues were RANK-positive^9^ and scattered reports showed that the role of RANK in CRC was related to the bone environment or osteoclast activity^14–16^, the direct function of RANK in CRC is still unknown. In this work, we clearly demonstrated positive immunostaining of RANK in CRC tissues, and confirmed high RANK expression in the CRC cell line HT29 and its low expression in SW480 and Caco2 cells. These results were similar to those reported by previous studies^4,9^. In addition, we found that RANK expression correlated negatively with the prognosis of patients with CRC. Gain and loss of function studies showed that RANK promoted the migration and invasion of CRC cells. Overexpression of RANK in CRC cells resulted in constitutive activation of the canonical downstream signaling pathway (p-P65, p-P38, p-AKT, and p-ERK). Moreover, presence of RANKL significantly increased the basal levels of these RANK downstream targets. Thus, our results clearly demonstrated that overexpression of RANK led to RANKL-dependent and RANKL-independent activation of downstream pathways, as described in other cancers^4,13^. Interestingly, we found that tumor-infiltrating CD25^+^FOXP3^+^Treg cells were the major producers of RANKL within the microenvironment of RANK-expressing CRC, which may provide a reference for the treatment of CRC metastasis. Taken together, our study proved that RANK expressed in CRC is functional.

Overexpression of RANK promoted CRC metastasis by activating the Ca^2+^-calcineurin/NFATC1-ACP5 axis. Previous studies have proven that osteoclast-associated signaling molecules promote CRC metastatic capacity, notably the transcription factor NFATC1 and its regulation by calcineurin^38,39^. Questions concerning which factor activates the calcineurin/NFATC1 axis in CRC and how calcineurin/NFATC1 regulates CRC metastasis remain unanswered. In the present study, we found that PLCγ-IP3-mediated ER Ca^2+^ release and STIM1-regulated Ca^2+^ influx through SOCE acted together to activate calcineurin/NFATC1 in *RANK*-overexpressing CRC cells. Moreover, we found that STIM1 was also regulated by PLCγ, indicating that PLCγ-IP3 signaling and STIM1 were involved closely in the Ca^2+^ oscillation induced by RANK in CRC. After being activated, calcineurin enhances NFATC1 activity by dephosphorylating it, thus increasing NFATC1’s nuclear translocation. NFATC1 further activates the transcription of the osteoclast-specific gene, *ACP5*. ACP5 is a metalloenzyme of the acid phosphoprotein phosphatase family^40^ and the major function of ACP5 in bone resorption is the catabolic degradation of bone matrix phosphoproteins. ACP5 has been recommended as a serum marker for bone resorptive activity in pathological states, such as osteoporosis and notably, bone metastasis of cancers^41,42^. Previous studies revealed that high ACP5 expression correlated with reduced survival and increased metastasis in various cancers^43–45^. In addition, ACP5 has been proven to aggravate the proliferation and invasion of CRC cells and might serve as an indicator for poor prognosis in CRC^18^. Consistently, our research also showed that RANK-induced ACP5 affected the NFATC1 pathway to promote the migration and invasion of CRC cells significantly. These results support a role for RANK in the regulation of CRC metastasis and provide novel insights into the molecular pathways linking RANK expressed in CRC to osteoclast differentiation signaling. Therefore, inhibition of osteoclastogenesis signaling not only interrupts the vicious cycle between bone and CRC, but also more importantly, it directly decreases the metastatic ability of CRC with high RANK expression. Moreover, these results implied that OPG and denosumab, the drugs that block RANKL–RANK interaction, are not sufficient to suppress CRC bone metastasis and skeletal-related events (SREs). Drugs targeting the Ca^2+^-calcineurin/NFATC1-ACP5 axis in RANK-expressing CRC might be needed, such as the inhibitors CsA, 2APB, and U73122 used in the present study.

There were several limitations of this study. Although CRC metastasis was independent of exogenous RANKL, the presence of RANKL could enhance *RANK*-overexpressing CRC metastasis and RANK signaling activation. Our IHC staining found that RANKL might be derived from tumor-infiltrating CD25^+^FOXP3^+^ Treg cells. Further experiments are needed to verify the exact source of RANKL to better determine the role of the RANK–RANKL system in CRC. In addition, it remains to be determined how RANK initially activates PLCγ to induce the Ca^2+^-calcineurin/NFATC1 axis, which will require further mechanistic experiments.

In conclusion, our findings demonstrated that RANK is upregulated in CRC and correlates with poor outcomes in patients with CRC. In addition, we determined a novel role of RANK expression in promoting CRC metastasis in vitro and in vivo. Mechanistically, in CRC, RANK induces PLCγ-IP3-STIM1 signaling-mediated Ca^2+^ oscillation and further activated the calcineurin/NFATC1-ACP5 pathway to regulate CRC metastasis. This axis linking RANK to osteoclast-specific gene *ACP5* might provide promising targets to develop antimetastatic agents to treat patients with CRC.

## Materials and methods

### Patients and tissue specimens

A total of 203 pairs of CRC and nontumor colonic epithelium paraffin-embedded tissues were obtained from operated patients at The First Affiliated Hospital, Sun Yat-sen University (Guangzhou, China) from 2008 to 2011. Follow-ups of these 203 patients ended on December 1st, 2019. The follow-up duration ranged from 2 to 130 months. The fresh CRC and matched normal tissues were collected from operated patients at The Seven Affiliated Hospital, Sun Yat-sen University (Shenzhen, China). Patients who underwent preoperative chemotherapy and/or radiotherapy were excluded. Each patient provided written informed consent. The use of clinical samples was approved by the Ethics Committee of Sun Yat-sen University.

### Antibodies and reagents

Primary antibodies used in this study included: anti-RANK (ab13918), anti-FOXP3 (ab20034), anti-CD3 (ab16669), anti-CD19 (ab134114), anti-rabbit RANKL (ab9957), anti-CD25 (ab128955), anti-CD4 (ab133616), anti-CD8 (ab4055), and anti-CD68 (ab201340) from Abcam; anti-ERK (#9102), anti-p-ERK (Thr202/Tyr204; #9101), anti-P38 (#9212), anti-p-P38 (Thr180/Tyr182; #9211), anti-P65 (#4764), anti-p-P65 (Ser536; #3033), anti-AKT (#9272), and anti-p-AKT (Ser473; #9101) from CST; anti-IgG isotype control (10500 C) from Thermo; anti-GAPDH (60004-1-lg), anti-STIM1 (11565-1-AP), GATA3 (66400-1-lg), and anti-mouse RANKL (66610-1-lg) from Proteintech, Wuhan, China; anti-ACP5 (DF6989), anti-rabbit RORγ (DF3196), and anti-T-bet (DF7759) from Affinity Biosciences, OH, USA; anti-mouse RORγ (sc-365476) from Santa Cruz, CA, USA; and anti-lamin B1 from (AF1408, Beyotime, Beijing, China). The reagents included: CsA, U73122, 2APB, and ionomycin from MCE; RANKL, EGF, and bFGF from PeproTech; and denosumab from TheraMabs (Shanghai, China).

### Immunohistochemistry

After routine deparaffinization and rehydration, treatment with hydrogen peroxide, antigen retrieval, and blocking with goat serum, the tissue sections were incubated with primary antibodies overnight at 4 °C. Rabbit IgG was used as negative control. All antibodies were diluted according to the instructions. Next, the slides were incubated with secondary antibody, stained with diaminobenzidine tetrahydrochloride (DAKO, Carpinteria, CA), and counterstained with hematoxylin. The IHC staining was analyzed by two independent pathologists who were blinded to the patient’s clinical parameters. The staining intensity was documented as 0 (no staining), 1 (weak immunoreactivity), 2 (moderate immunoreactivity), and 3 (strong immunoreactivity). The percentage of immunoreactive cells was scored as 0 (no positive cells), 1 (<20%), 2 (20–50%), 3 (51–75%), and 4 (>75%). The final score was determined by multiplying the intensity and the percentage of immunoreactive cells. Depending on the final score, the staining results were graded as − (score 0), + (score 1–4), ++ (score 5–8), and +++ (score 9–12). The grade was furthermore defined as low expression (−, +) and high expression (++, +++)^46,47^.

### Cell lines and culture conditions

Human CRC cell lines SW480, Caco2, HT29, DLD1, HCT116, LS174T, and LOVO were purchased from the American Type Culture Collection. All cells were authenticated and free of mycoplasma. HT29 were cultured in RPMI 1640 Medium (Gibco), and the other cell lines were maintained in Dulbecco’s modified Eagle’s medium (Gibco) at 37 °C with 5% CO_2_. Medium was added with 10% fetal bovine serum (FBS; Gibco) for cell culture.

### Cell lines transfection

For transient transfection, two independent siRNA duplexes against *STIM1*, *ACP5*, and control siRNA, overexpression plasmids including pFV155-GFP-puro-*ACP5* and control vector were introduced into cells using Lipofectamine 3000 reagent (Invitrogen), according to the manufacturer’s instructions. For stable overexpression and knockdown transfection, 293T cells were transfected with pFV155-GFP-puro-*RANK*, pLKO.1-puro-*RANK*-target shRNA, PTSB-NEO-*NFATC1*-target shRNA, and their empty vectors using package plasmids and polyethyleneimine (Polysciences). Virus particles were harvested 48 h after transfection and used for cell infection with polybrene (Sigma). Puromycin (Sigma) and G418 (Sigma) were used for puro-resistance and NEO-resistance plasmids, respectively. All siRNA duplexes, shRNA constructs, and overexpressing plasmids were from Transheep (Shanghai, China). The siRNA and shRNA sequences are listed in Supplementary Tables S1 and S2.

### Western blot analysis

Briefly, proteins from total cell lysates (20 μg) were separated by SDS–PAGE, transferred onto polyvinylidene difluoride membranes (Millipore), and immunoblotted with the indicated antibodies, followed by peroxidase-conjugated anti-mouse or rabbit IgG. Anti-GAPDH antibody was diluted at 1:10,000 and other primary antibodies were 1:1000. Nuclear and Cytoplasmic Protein Extraction kit (Beyotime, Beijing, China) was used to separate the nuclear and cytoplasmic protein, according to the manufacturer’s protocol. Membranes were finally infiltrated by an enhanced chemiluminescence reagent and visualized using Chemiluminescence imaging Systems (Bio-Rad).

### Quantitative reverse transcription polymerase chain reaction

Total RNA was extracted from cells using AG RNAex Pro Reagent (AG21102, ACCURATE BIOTECHNOLOGY, Hunan, China), according to the manufacturer’s protocol. Reverse transcription was performed using the Evo M-MLV RT Premix (AG11706, ACCURATE BIOTECHNOLOGY, Hunan, China). The qRT-PCR assay was performed using SYBR^®^ Green Premix Pro Taq HS qPCR Kit (AG11701, ACCURATE BIOTECHNOLOGY, Hunan, China) in the CFX96 Touch Real-Time PCR system (CFX96, BIO-RAD Laboratories, Hercules, USA). *GAPDH* was used as an endogenous reference to normalize RNA expression. The primer sequences used are listed in Supplementary Table S3.

### Migration and invasion assays

A total of 2 × 10^5^ cells in serum-free medium were seeded into the upper chamber of inserts (8 μm pore size, BD Biosciences, USA) with or without diluted Matrigel (Corning, USA) for migration and invasion in 24-well plates. Besides, the indicated concentration of chemicals used in this study was added into the upper chambers to evaluate their effect. Medium supplemented plus 20% FBS was used as a chemoattractant in the lower chambers. After 60-h incubation, the cells on the lower membrane were fixed and stained with crystal violet. The staining cells were randomly imaged in eight different fields with a microscope.

### Cell proliferation assay

A total of 1000 cells were inoculated into 96-well plates with complete medium. Four consecutive days after initial planting, cells were incubated in the medium with 10% Cell Counting Kit-8 (Dojindo, Japan) for 2 h at 37 °C in dark. The cell absorbance was then detected at 450 nm with a microplate reader (Synergy H1M, BioTek).

### Immunofluorescence

Cells were seeded onto sterile slides into 24-well culture plates. After reached a suitable confluence, cells were fixed with 4% paraformaldehyde, permeabilized with 0.1% Triton/PBS, and blocked with goat serum. The methods for tissue slices before primary antibody incubation were the same as IHC.

Next, cells were incubated with indicated primary antibody overnight at 4 °C. For double immunofluorescence of tissue slices, both primary antibodies were mixed and incubated. All antibodies were used according to the instructions. On the second day, cells or slices were further incubated with Alexa Fluor 488 goat anti-mouse IgG (A-11001, Thermo) or Alexa Fluor 594 goat anti-rabbit IgG (A-11012, Thermo), stained with DAPI (Sigma) label nuclei, and finally observed under Leica fluorescence or Zeiss confocal microscopy.

### Animal experiments

Female 6-week-old BABL/c nude mice were purchased from Beijing Vital River Laboratory Animal Technology Company (Beijing, China). All animal experiments were approved by the Sun Yat-sen University Institutional Animal Care and Use Committee. The mice were randomly allocated into two groups (*n* = 6) and received intrasplenic transplantation to study liver metastasis. For the spleen injection, the mice were anesthetized with isoflurane and laparotomized to expose the spleen. A total of 1.5 × 10^6^ SW480-Luc/Control or SW480-Luc/RANK cells in 50 μl PBS were slowly injected into the spleen using an insulin syringe. The spleen was then replaced into the abdomen cavity and the abdominal wall was sutured. Liver metastasis was determined by bioluminescence imaging using the IVIS Imaging System (Xenogen). After 15 weeks, the mice were euthanized and the livers were removed for bioluminescence imaging and HE staining to confirm metastatic foci. The statistical analyses were performed by researchers blinded to experiment design.

### Calcium-flux measurements

Cells were trypsinized and incubated with 10 μM Calbryte™ 630 AM Esters (AAT Bioquest) for 1 h at room temperature, according to instruction. Then, cells were switched to Hanks and Hepes buffer containing 2 mM Ca^2+^, and subjected to FACS analysis by a CytoFLEX flow cytometer (Beckman) to measure fluorescence intensity. During the FACS analysis, cells were stimulated with ionomycin (2 μg/ml). The results were further quantitatively analyzed by FlowJo (v10) software.

### Bioinformation analysis

The gene expression data and Kaplan–Meier survival plots for CRC patients were appraised by online database GEPIA (Gene Expression Profiling Interactive Analysis, http://gepia.cancer-pku.cn/), UALCAN (http://ualcan.path.uab.edu/analysis.html), and Prognostic Database (http://genomics.jefferson.edu/proggene/). The mRNA expressions of *RANK* in CRC cell lines were analyzed using the CCLE database (https://portals.broadinstitute.org/ccle/home). Correlation analysis between gene expression in tissues of CRC patients was computed from cBioPortal (www.cbioportal.org), TIMER (Tumor IMmune Estimation Resource, https://cistrome.shinyapps.io/timer/), and GEPIA. CHIP-seq datasets were collected from the ENCODE Project Consortium (https://www.encodeproject.org/).

### Statistical analysis

Statistical analysis was performed using SPSS 20.0 and GraphPad Prism 6.0. The indicated protein levels to GAPDH are quantified using ImageJ software. Data were compared by Student’s *t* test or one-way ANOVA test. The *χ*^2^ test and Fisher’s exact test were used to analyze the relationship between RANK expression and clinical characteristics. Survival curves were plotted with the Kaplan–Meier method with the log-rank test. Cox regression was employed for univariate and multivariate analyses. Variables with a *P* value < 0.05 in univariate analysis were included in multivariate analysis. All data are presented as the mean ± SD. *P* value < 0.05 was considered statistically significant.

## Supplementary information

Table S1

Table S2

Table S3

Supplementary figure legends

Figure.S1

Figure.S2

Figure.S3

Figure.S4

Figure.S5

Figure.S6

Figure.S7

Figure.S8
