# Supplementary material for: RANK promotes colorectal cancer migration and invasion by activating the Ca2+-calcineurin/NFATC1-ACP5 axis
Source: Cell Death Dis. 2021 Apr 1;12(4):336. doi: 10.1038/s41419-021-03642-7 (PMC8016848; doi:10.1038/s41419-021-03642-7)
Supplement: Supplementary file 1 — Table S1 [file 41419_2021_3642_MOESM1_ESM.docx]

**Table S1.** **Target sequences of siRNA**

| Name | Target sequences |
| --- | --- |
| si-STIM1-1 | CUCUCUUGACUCGCCAUAA |
| si-STIM1-2 | ACAGUGGCUGAUCACAUAU  CGGGCAAGUCCCUCUUUAA  CAAUGUCUCUGCCCAGAUU |
| si-ACP5-1  si-ACP5-2 |  |
