# Supplementary material for: RANK promotes colorectal cancer migration and invasion by activating the Ca2+-calcineurin/NFATC1-ACP5 axis
Source: Cell Death Dis. 2021 Apr 1;12(4):336. doi: 10.1038/s41419-021-03642-7 (PMC8016848; doi:10.1038/s41419-021-03642-7)
Supplement: Supplementary file 2 — Table S2 [file 41419_2021_3642_MOESM2_ESM.docx]

**Table S2.** **Target sequences of shRNA**

| Name | Target sequences |
| --- | --- |
| sh-TNFRSF11A-1 | GATAAATGCTTGCTGCATAAA |
| sh-TNFRSF11A-2  sh-NFATC1-1  sh-NFATC1-2 | TGTTTACTTGCCCGGTTTAAT  CCCGCCAACGTTCCAATTATA  ATTTGCTACTGTAGGAGTATT |
