# Supplementary material for: RANK promotes colorectal cancer migration and invasion by activating the Ca2+-calcineurin/NFATC1-ACP5 axis
Source: Cell Death Dis. 2021 Apr 1;12(4):336. doi: 10.1038/s41419-021-03642-7 (PMC8016848; doi:10.1038/s41419-021-03642-7)
Supplement: Supplementary file 3 — Table S3 [file 41419_2021_3642_MOESM3_ESM.docx]

**Table S3.** **Sequences of primers**

| Name |  | Sequences |
| --- | --- | --- |
| GAPDH | Forward | 5'-GTCTCCTCTGACTTCAACAGCG-3' |
|  | Reverse | 5'-ACCACCCTGTTGCTGTAGCCAA-3' |
| STIM1 | Forward | 5'-CACTCTTTGGCACCTTCCACGT-3' |
|  | Reverse | 5'-CTGTCACCTCGCTCAGTGCTTG-3' |
| ORAI1 | Forward | 5'-AGGTGATGAGCCTCAACGAGCA-3' |
|  | Reverse | 5'-AGTCGTGGTCAGCGTCCAGCT-3' |
| TRPC1 | Forward | 5'-CCAAACTGCTGGTGGCAATGCT-3' |
|  | Reverse | 5'-GGAATGATGTTGAAAGGTGGAGG-3' |
| NFATC1 | Forward | 5'-CACCAAAGTCCTGGAGATCCCA-3' |
|  | Reverse | 5'-TTCTTCCTCCCGATGTCCGTCT-3' |
| MMP9 | Forward | 5'-GCCACTACTGTGCCTTTGAGTC-3' |
|  | Reverse | 5'-CCCTCAGAGAATCGCCAGTACT-3' |
| CTSK | Forward | 5'-GAGGCTTCTCTTGGTGTCCATAC-3' |
|  | Reverse | 5'-TTACTGCGGGAATGAGACAGGG-3' |
| ACP5 | Forward | 5'-CATGACCACCTTGGCAATGTCTC-3' |
|  | Reverse | 5'-CTGTGGGATCTTGAAGTGCAGG-3' |
| VCAM1 | Forward | 5'-GATTCTGTGCCCACAGTAAGGC-3' |
|  | Reverse | 5'-TGGTCACAGAGCCACCTTCTTG-3' |
| RANK | Forward | 5'-GCTCAACAAGGACACAGTGTGC-3' |
|  | Reverse | 5'-CGCATCGGATTTCTCTGTCCCA-3' |
