# Supplementary material for: RANK promotes colorectal cancer migration and invasion by activating the Ca2+-calcineurin/NFATC1-ACP5 axis
Source: Cell Death Dis. 2021 Apr 1;12(4):336. doi: 10.1038/s41419-021-03642-7 (PMC8016848; doi:10.1038/s41419-021-03642-7)
Supplement: Supplementary file 4 — Supplementary figure legends [file 41419_2021_3642_MOESM4_ESM.docx]

**Fig. S1** Related to **Fig. 2** **RANK promoted the metastasis of CRC cells in vitro and in vivo.** **a** *RANK* mRNA expressions among CRC cell lines based on their RNA sequence from the CCLE database. **b, c** *RANK* overexpression or knockdown efficiency was confirmed by qRT-PCR in CRC cells. **d** The *RANK*-overexpressing CRC cells transduced with GFP vector expressed green fluorescent. Scales bars = 100 μm. **e, f** Cell proliferation of *RANK*-overexpressing CRC cells and control cells with or without 100 ng/ml RANKL was measured by CCK-8 assay. **g** IHC staining of RANK, RANKL, CD3, CD19, FOXP3, and CD25 in CRC tissue parallel sections. Scales bars = 100 μm (200×), 50 μm (400×). **h** Human CRC tissue sections were co-stained with FOXP3 and RANKL specific antibodies using immunofluorescence. Nuclear DNA was counterstained with DAPI. Scales bars = 50 μm. Data are mean ± SD (n = 3). *****P* < 0.0001

**Fig. S2** Related to **Fig. 2** **RANK promoted the metastasis of CRC cells in vitro and in vivo.** Serial human CRC tissue sections showing expression of RANKL, RANK, CD4, FOXP3, T-bet, GATA3, RORγT, CD68, CD8 by IHC staining. Shown is a representative lesion area. Scales bars = 200 μm (40×), 50 μm (200×). ***P* < 0.01, ****P* < 0.001, *****P* < 0.0001

**Fig. S3** Related to **Fig. 2** **RANK promoted the metastasis of CRC cells in vitro and in vivo.** Representative immunofluorescence images of co-staining for RANKL/CD4, RANKL/CD8, RANKL/RORγT, RANKL/T-bet, RANKL/GATA3, and RANKL/CD68 in human CRC tissue sections. RANKL was co-localized with CD4 (white). Cell nuclei were counterstained with DAPI (blue). Primary antibodies are indicated in the figures. Scales bars = 100 μm.

**Fig. S4** Related to **Fig. 2** **RANK promoted the metastasis of CRC cells in vitro and in vivo. a** Western blotting showing endogenous RANKL expression in CRC cells. The protein levels of RANKL are quantified using ImageJ software. **b, c** Representative photomicrographs and histograms illustrating the effect of denosumab (0, 1, 10, 100 μg/ml) on migration and invasion of SW480 and Caco2 cells.

**Fig. S5** Related to **Fig. 3** **RANK regulated CRC migration and invasion by activating ACP5 expression.** **a** Western blot analysis of phosphorylated ERK1/2, P38, AKT, and P65 in *RANK-* overexpressing CRC cells and control cells. **b** Representative immunoblots depicted increased activation of indicated signal transduction pathways upon RANKL treatment in SW480RK and Caco2RK cells compared with control groups. After starved for 24 hours, cells were then treated with 100 ng/ml RANKL and incubated for 0, 10, 20, 40 minutes. **c, d** QRT-PCR analysis for bone osteoclast markers (*ACP5*, *MMP9*, *CTSK*, and *VCAM1*) in *RANK*-overexpressing and knockdown CRC cells. *ACP5*, tartrate-resistant acid phosphatase. *CTSK*, cathepsin K. *MMP9*, matrix metalloproteinase 9. *VCAM1*, vascular cell adhesion molecule 1. **e** Detection of *ACP5* expression in CRC and normal tissues in GEPIA online database from TCGA data. **f** High *ACP5* expression predicted decreased overall survival in CRC patients by Kaplan-Meier analysis in Prognostic Database from GEO dataset (GSE17637). Data are mean ± SD (n = 3). **P* < 0.05, ***P* < 0.01, ****P* < 0.001, *****P* < 0.0001

**Fig. S6** Related to **Fig. 4 RANK upregulated ACP5 expression through driving NFATC1 nuclear translocation.** **a-d** Kaplan–Meier analysis of overall survival (OS) or relapse free survival (RFS) according to online dataset records of *NFATC1* expression in CRC patients. **e-g** Correlation plots of *RANK* and *NFACT1* in CRC patients from online databases. **h, i** QRT-PCR analysis of *NFATC1* in *RANK*-overexpressing and knockdown CRC cells. Data are mean ± SD (n = 3). ***P* < 0.01, ****P* < 0.001

**Fig. S7** Related to **Fig. 6 RANK activated calcineurin/NFATC1 axis by STIM1-mediated Ca^2+^ influx. a, b** The regulation of three major SOCE members by RANK in CRC cells by qRT-PCR analysis. *STIM*, stromal interaction molecule; *TRPC*, transient receptor potential channel. **c** The positive correlation between the mRNA expressions of *RANK* and *STIM1* based on the online dataset in CRC. **d-f** The indicated online databases illustrated a significantly positive correlation between mRNA expressions of *STIM1* and *ACP5* in CRC. Data are mean ± SD (n = 3). **P* < 0.05, ***P* < 0.01, ****P* < 0.001, *****P* < 0.0001

**Fig. S8** Related to **Fig. 7 RANK induced STIM1-mediated Ca^2+^ influx and ER Ca^2+^ release by activating the PLCγ-IP3 axis.** **a-d** The mRNA expressions of *RANK* were positively correlated with *IP3R* and *PLCγ* in CRC from the online databases. *PLCG2*, *PLCγ*. **e-g** Significant positive correlations existed between mRNA levels of *IP3R* and *STIM1* in CRC from the online databases. *ITPR3*, *IP3R*.
