# Supplementary figures and images for: RANK promotes colorectal cancer migration and invasion by activating the Ca2+-calcineurin/NFATC1-ACP5 axis
Source: Cell Death Dis. 2021 Apr 1;12(4):336. doi: 10.1038/s41419-021-03642-7 (PMC8016848; doi:10.1038/s41419-021-03642-7)

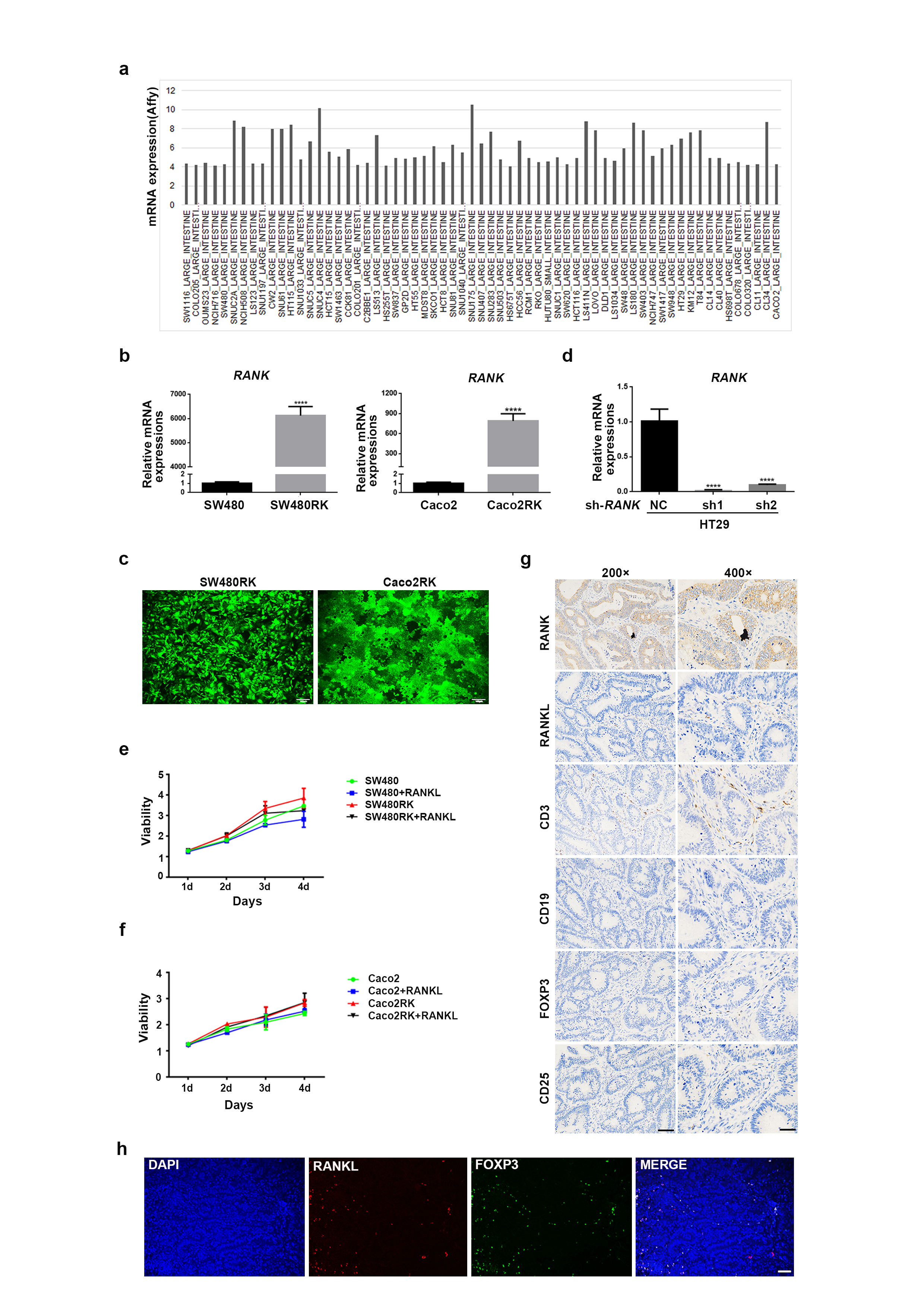

Supplement: Supplementary file 5 — Figure.S1 [file 41419_2021_3642_MOESM5_ESM.tif]

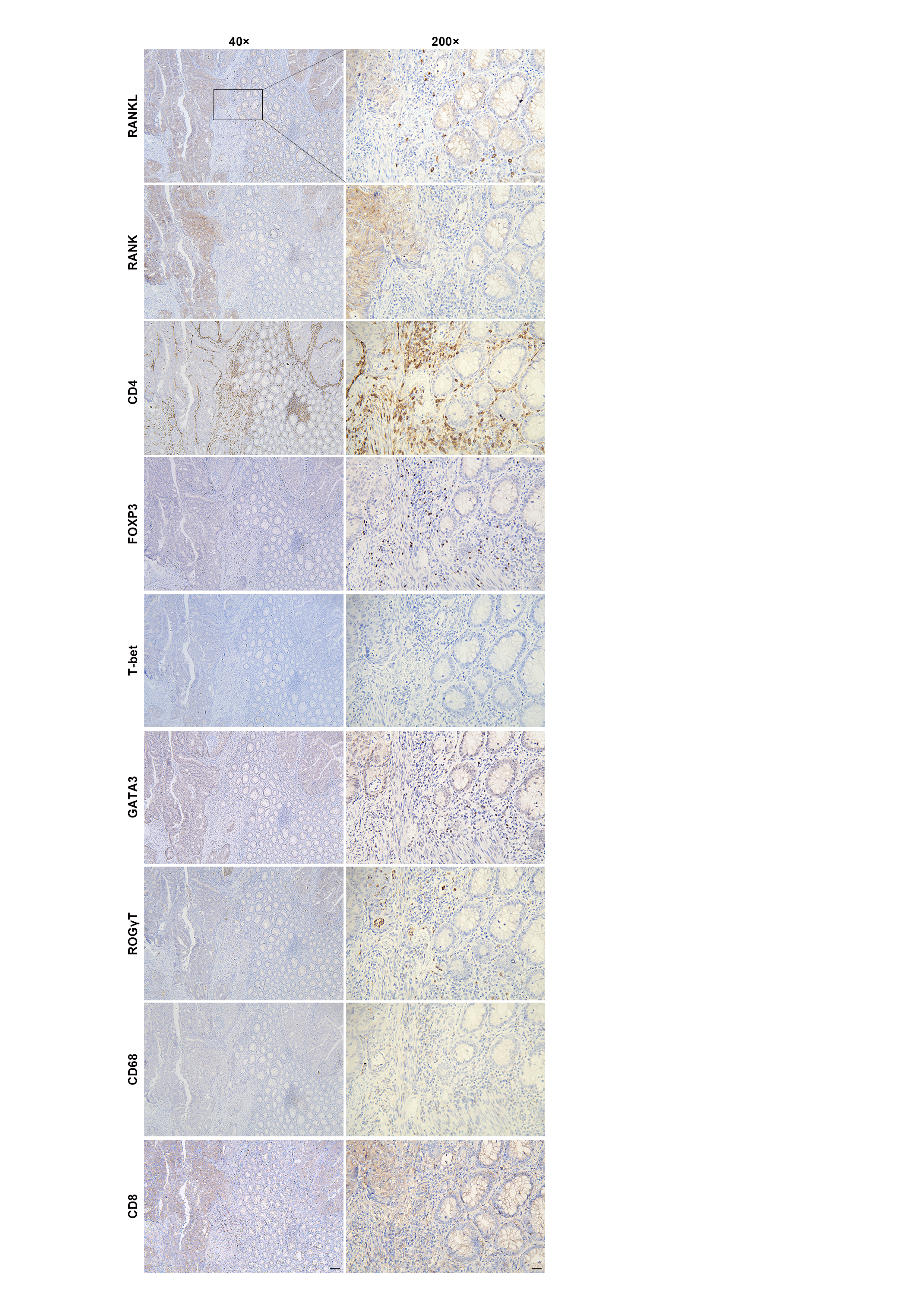

Supplement: Supplementary file 6 — Figure.S2 [file 41419_2021_3642_MOESM6_ESM.tif]

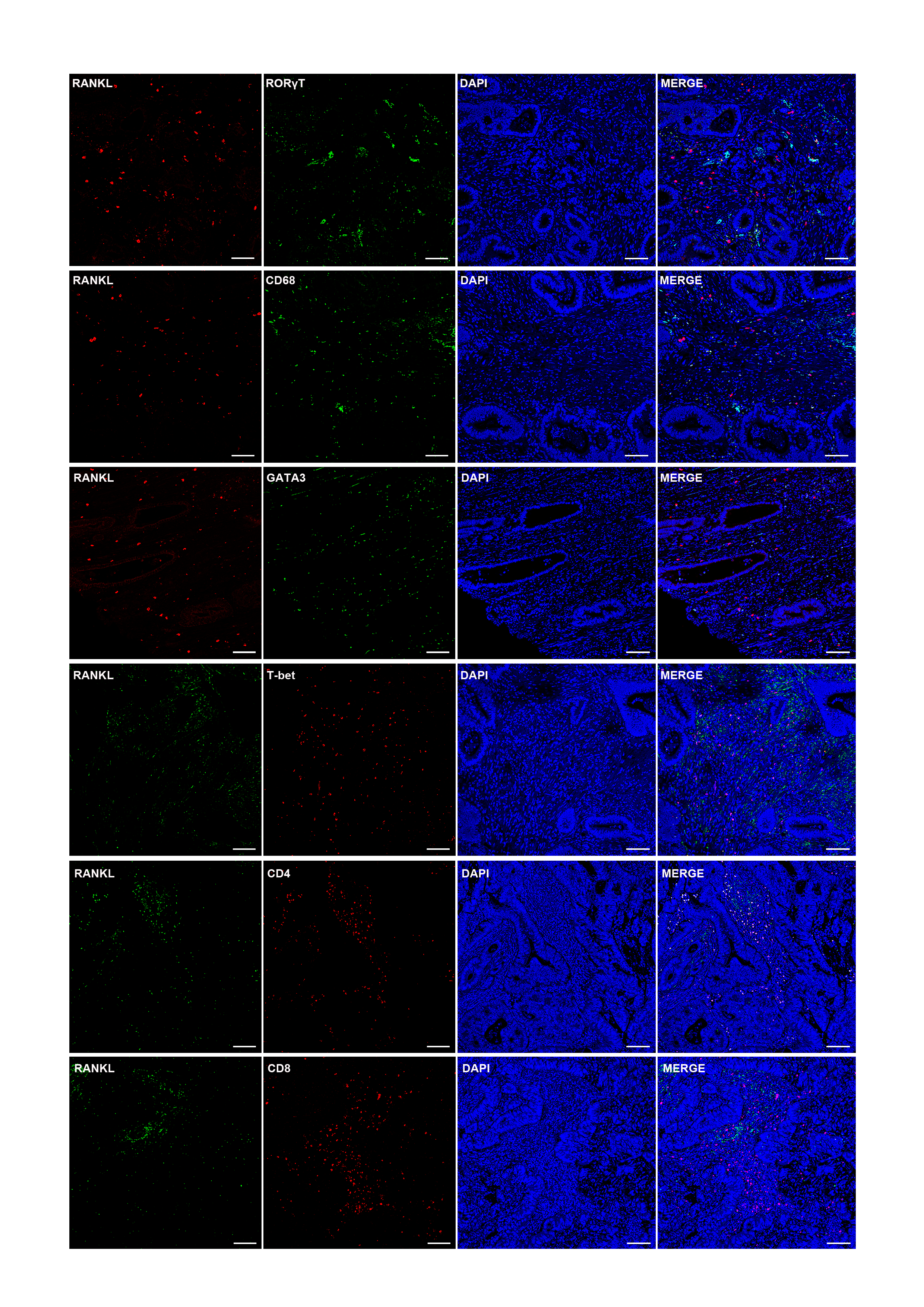

Supplement: Supplementary file 7 — Figure.S3 [file 41419_2021_3642_MOESM7_ESM.tif]

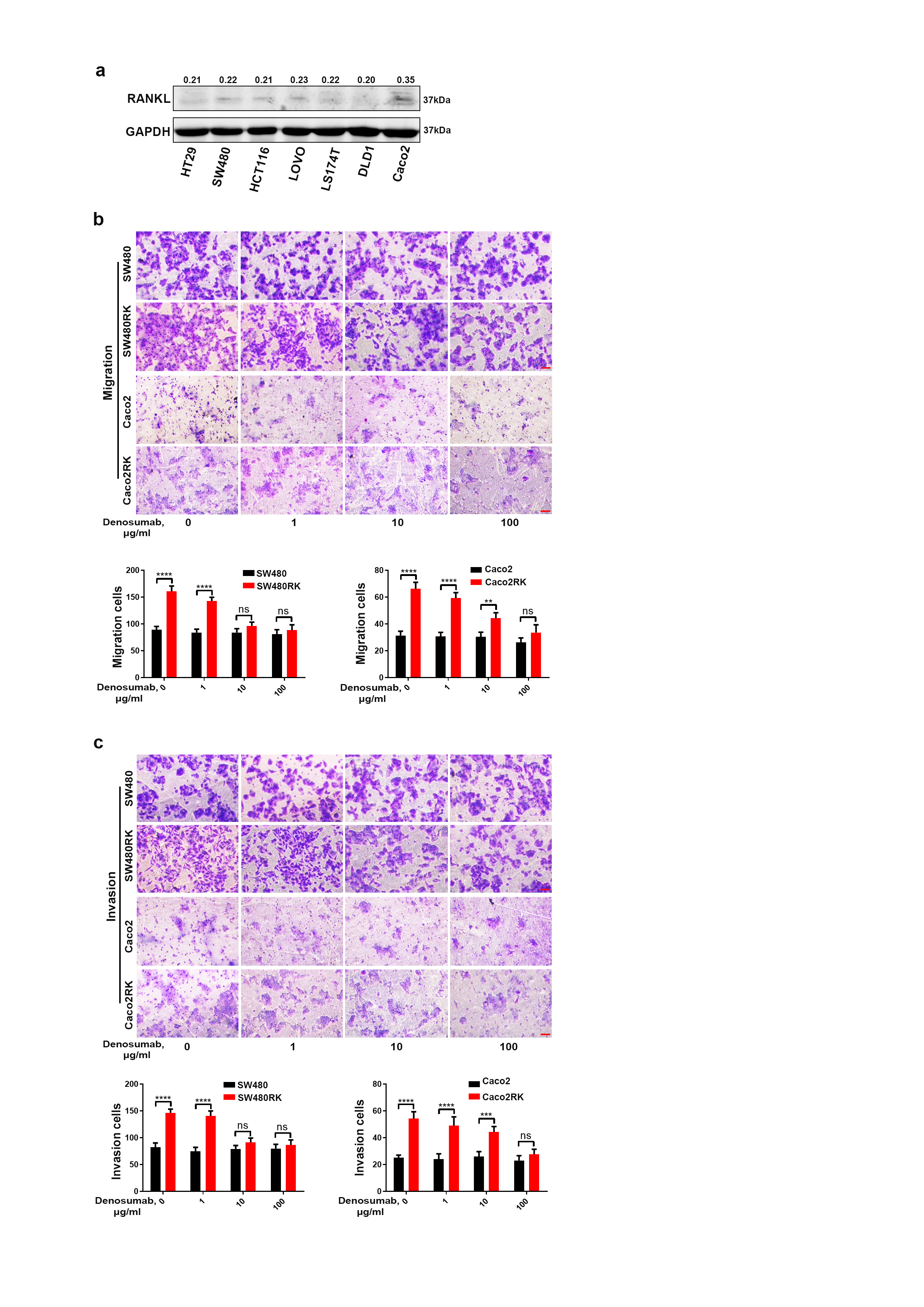

Supplement: Supplementary file 8 — Figure.S4 [file 41419_2021_3642_MOESM8_ESM.tif]

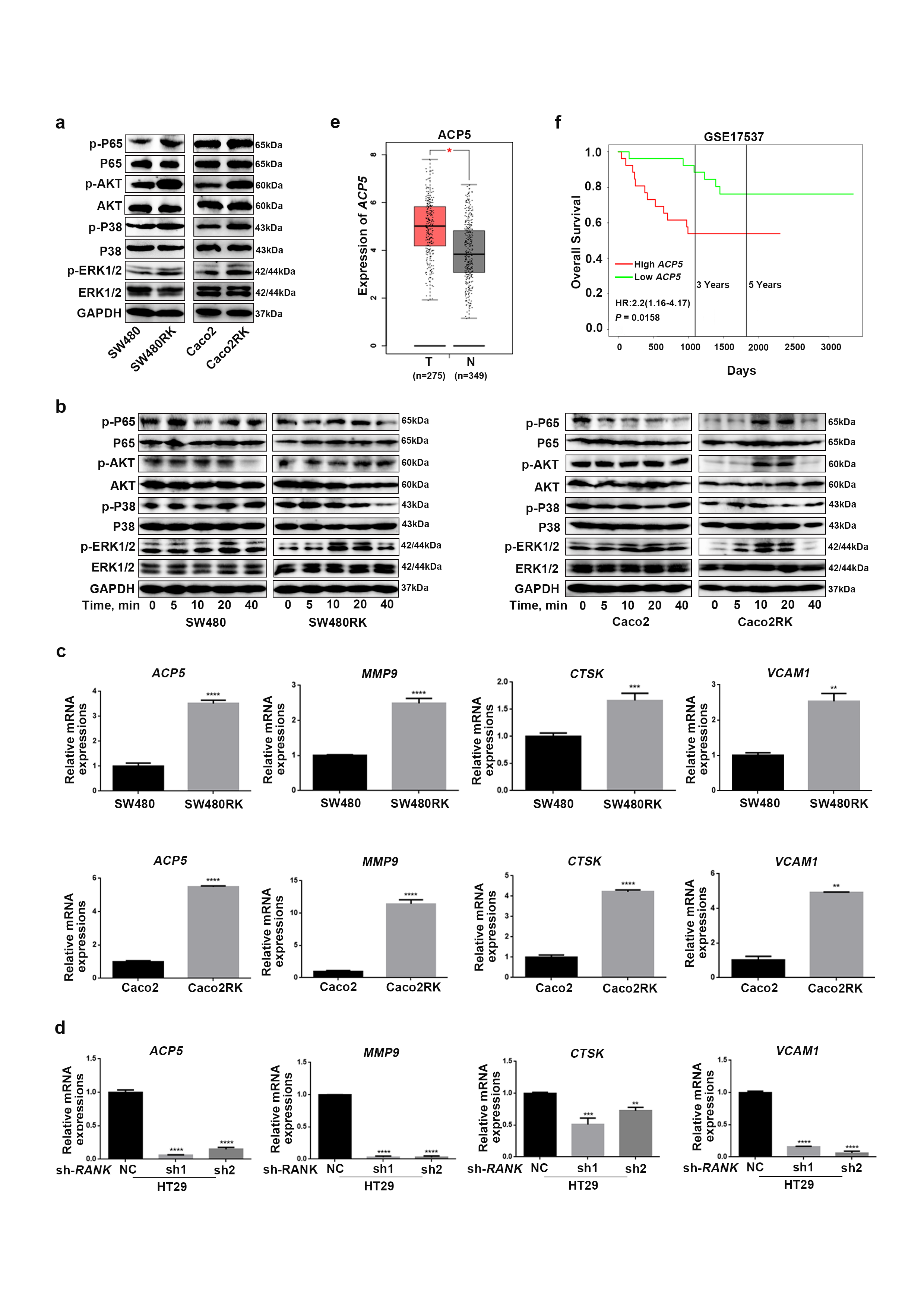

Supplement: Supplementary file 9 — Figure.S5 [file 41419_2021_3642_MOESM9_ESM.tif]

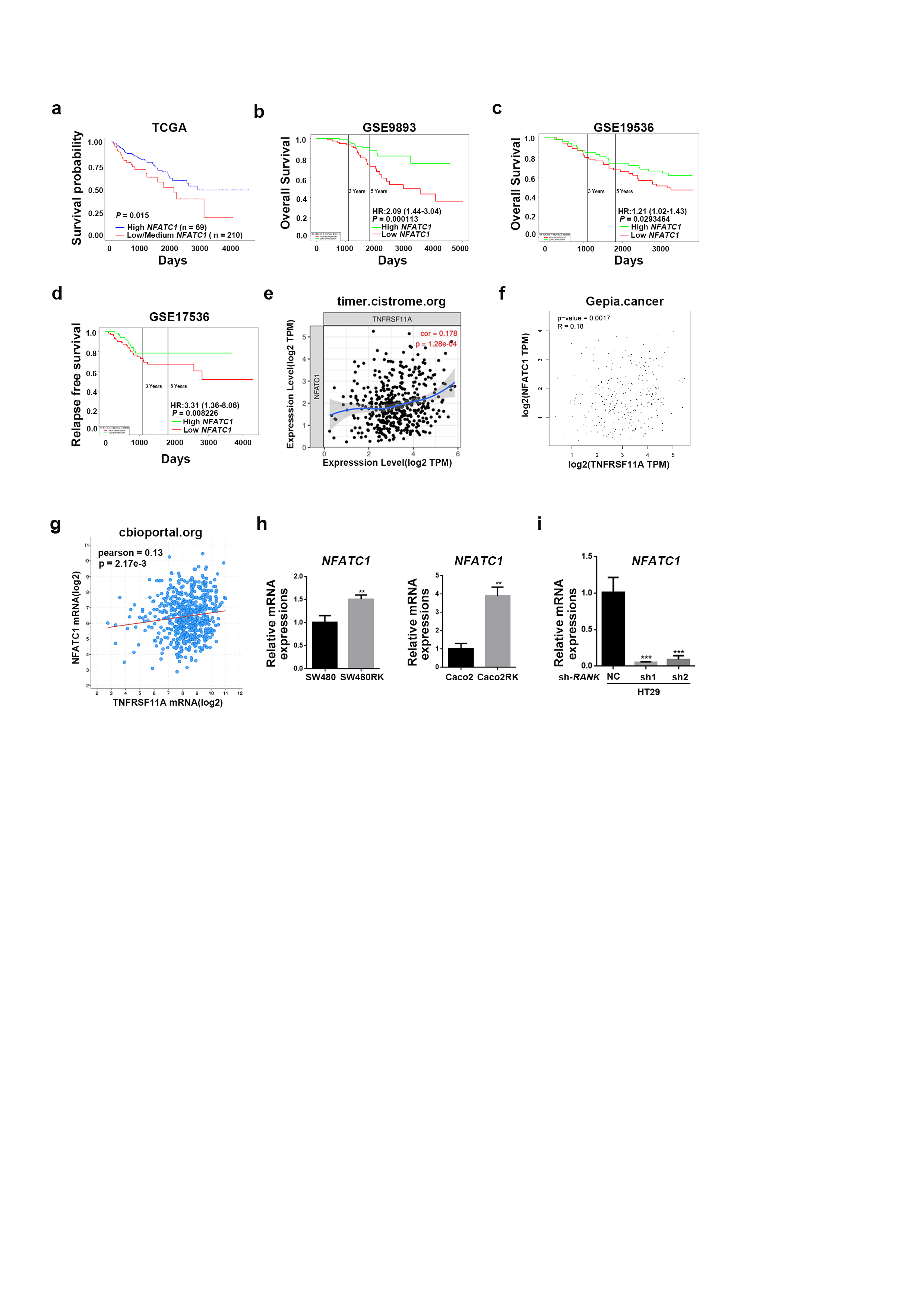

Supplement: Supplementary file 10 — Figure.S6 [file 41419_2021_3642_MOESM10_ESM.tif]

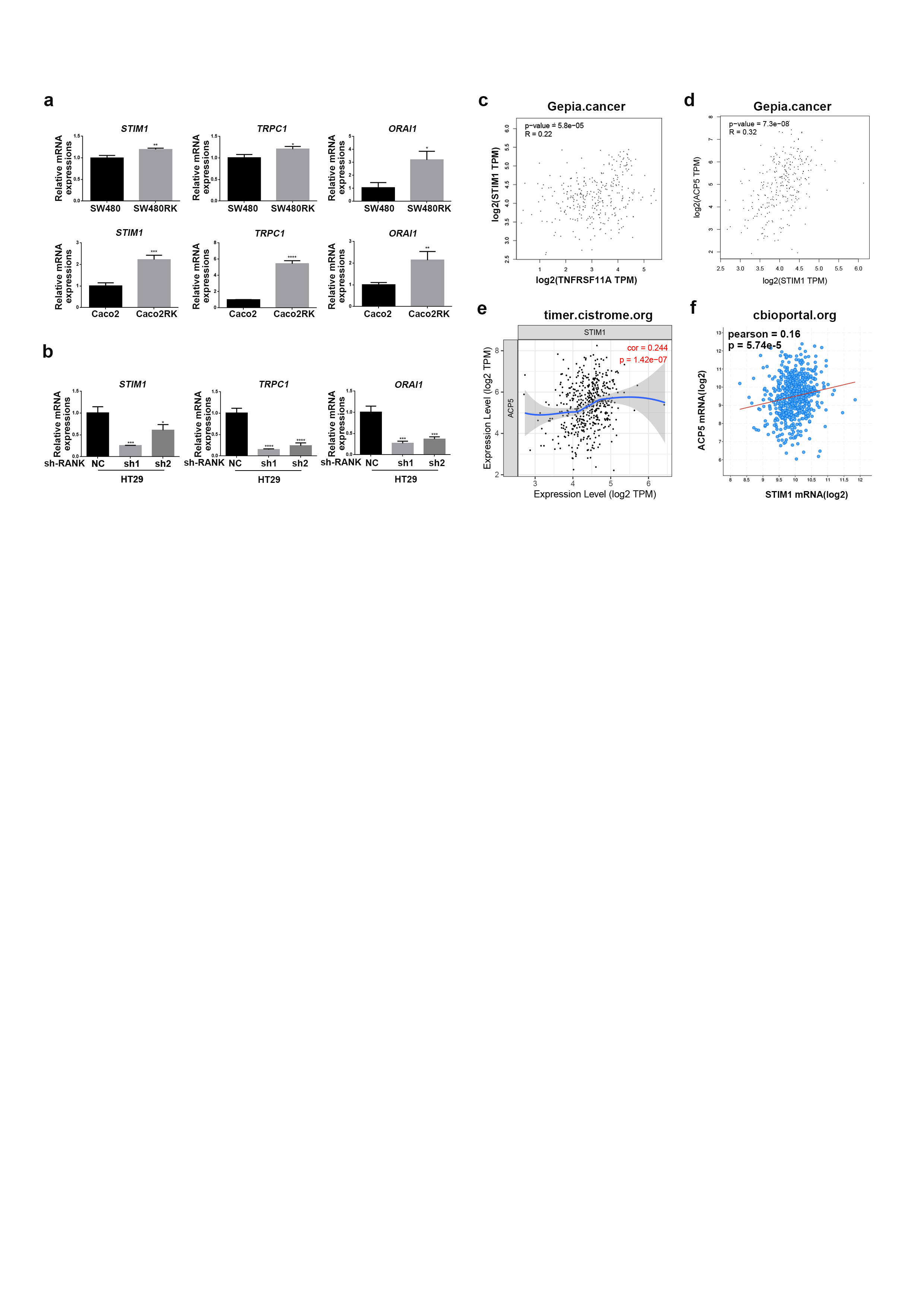

Supplement: Supplementary file 11 — Figure.S7 [file 41419_2021_3642_MOESM11_ESM.tif]

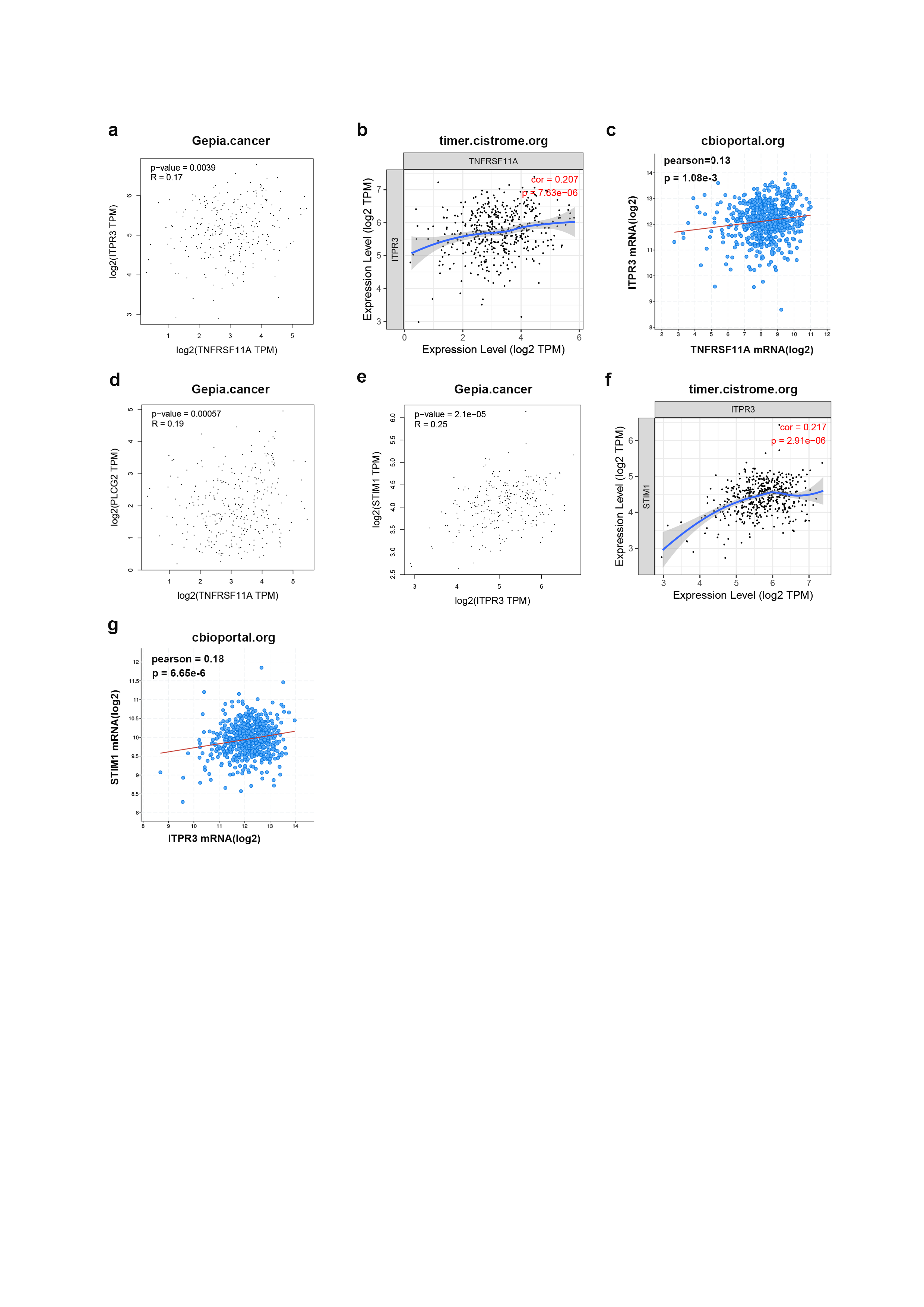

Supplement: Supplementary file 12 — Figure.S8 [file 41419_2021_3642_MOESM12_ESM.tif]
